# Supplementary material for: Wnt signalosome assembly is governed by conformational flexibility of Axin and by the AP2 clathrin adaptor
Source: Nat Commun. 2025 May 21;16:4718. doi: 10.1038/s41467-025-59984-9 (PMC12095580; doi:10.1038/s41467-025-59984-9)
Supplement: Supplementary file 1 — Supplementary Information [file 41467_2025_59984_MOESM1_ESM.pdf]

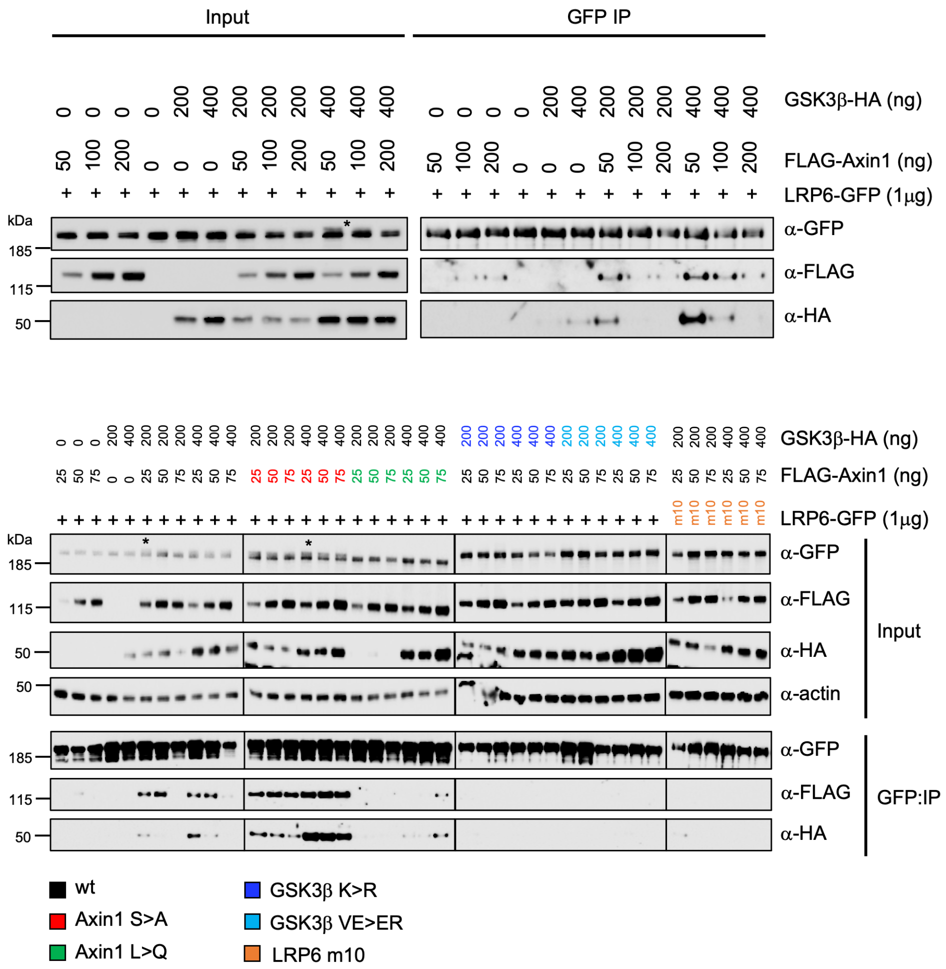

Supplementary Fig. 1. Optimization of coIP assays
Optimization of coIP assays, by titrating the amounts of the co-transfected wt (*black*) and mutant (*colored*) plasmids in the transfection mixtures, as indicated (color key below panel); shown are Western blots probed with antibodies (α) as indicated on the right following immunoprecipitation (IP); positions of molecular weight markers indicated on the left (in kDa).

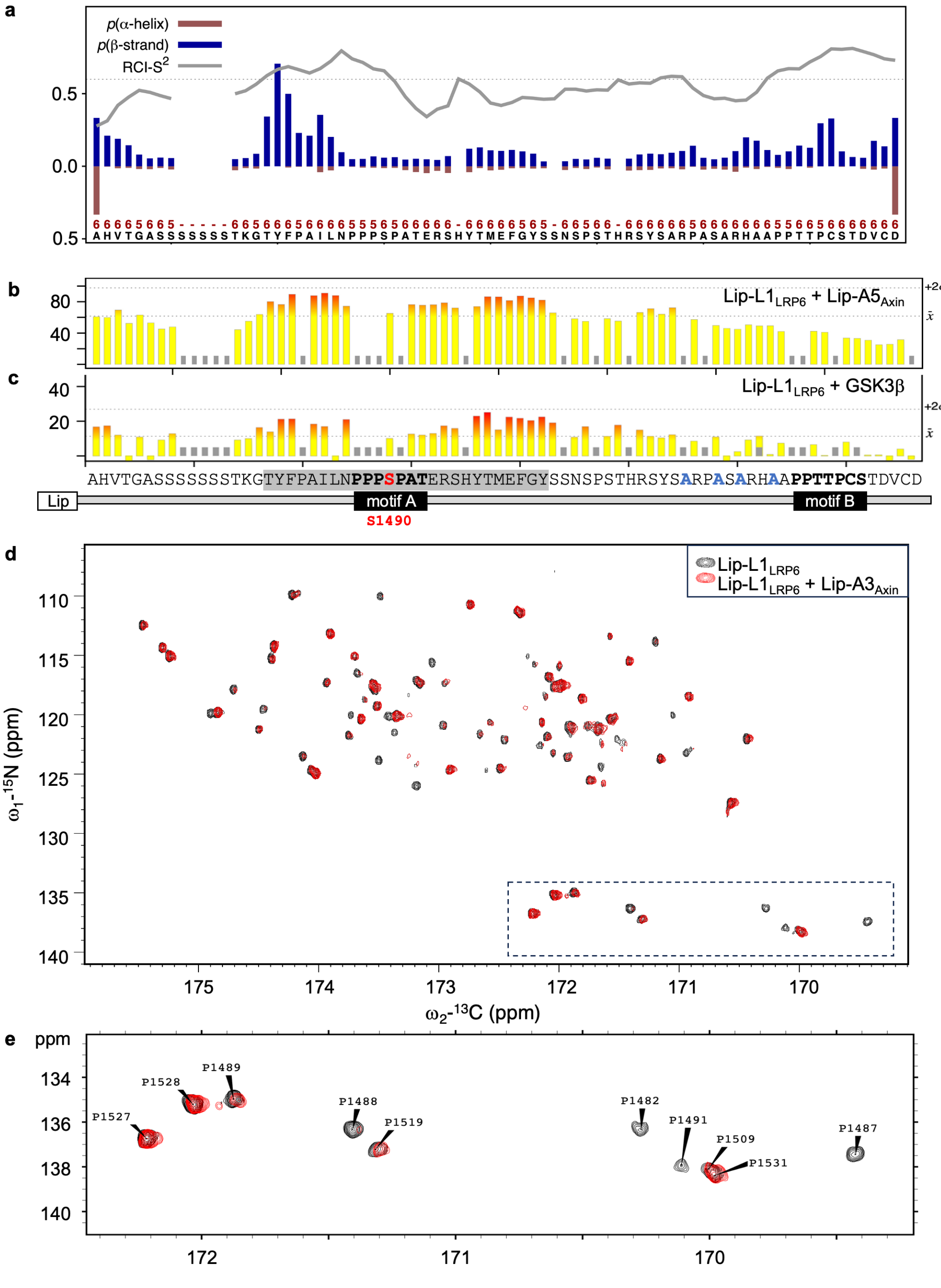

Supplementary Fig. 2. Binding sites for Axin- and GSK3 in the proximal LRP6 ctail
(**a**) TALOS-N plot for L1$_{LRP6}$ (LRP6$_{1463\text{-}1538}$ bearing YYYF>A; see **Fig. 2**, *bottom*), depicting the probability (*p*) of secondary structure (α-helix, *brown*; β-strand, *blue*) and the Random Coil Index order parameter (RCI-S^2, *gray*) which scales between 0 (total disorder) and 1 (fully structured); numbers above L1$_{LRP6}$ sequence (shown at the bottom) indicate numbers of chemical shift values for (HN,N,CA,CB,CO,HA). Of note, RCI-S^2 values <0.6 (indicated by dotted line) are considered dynamic, while values for stable α-helices are typically >0.9. (**b-c**) Bleach maps generated from BEST-TROSY spectra from 80 µM ^{15}N-labeled Lip-L1$_{LRP6}$ incubated with 80 µM (**b**) Lip-A5$_{Axin}$ or (**c**) GSK3β; bleach maps depict percentage attenuation of peak height by incubation with ligand ranging from strong (*red*) to weak (*yellow*); mean attenuations ($\bar{x}$) and means + 2 standard deviations (+2σ) are given on the right in this and subsequent bleach maps; gray bars indicate residues for which NMR peaks are not assigned or obscured by overlap. (**d, e**) Overlay of ^{13}C-detected 2D-CON spectra of 150 µM ^{15}N-^{13}C double-labeled-labeled Lip-L1$_{LRP6}$ by itself (*black*) or incubated with 150 µM Lip-A3$_{Axin}$ (*red*); see main **Fig. 2e**, for corresponding bleach map; color key above panels. (**e**) Expansion of the 134-138 ppm ^{15}N region of spectrum in (**d**), visualizing bleaching of proline residues which are not observable by BEST-TROSY.

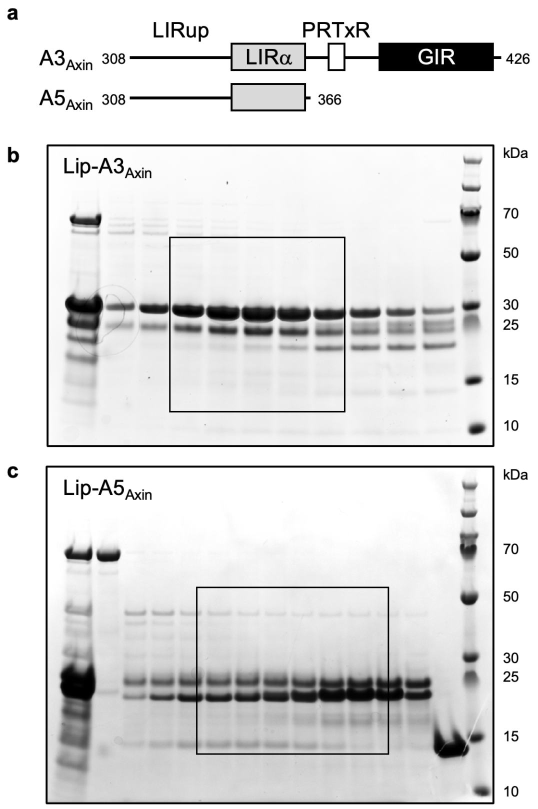

Supplementary Fig. 3. Sample preparations of $A3_{Axin}$ and $A5_{Axin}$ proteins
(**a**) Cartoons of Axin Lip-$A3_{Axin}$ and Lip-$A5_{Axin}$ (with numbers indicating 5' and 3' amino acids of human Axin1), with LRP6- and GSK3-interacting regions indicated (LIRup, LIRα, bipartite LRP6-interacting region; GIR, GSK3-binding region; PRTxR, contributes to multipronged interaction with GSK3). (**b, c**) SDS-PAGE analysis of representative sample preparations of (**b**) Lip-$A3_{Axin}$ and (**c**) Lip-$A5_{Axin}$; shown are total cleared lysates after elution from Ni-NTA agarose (*left*) and fractions obtained after subsequent size exclusion chromatography with a HiLoad 26/600 Superdex 75 pg column (see Methods); fractions pooled for subsequent analysis by NMR or ITC are boxed; *right*, molecular weight markers (in kDa). Note the partial degradation of samples which inevitably occurred during protein purification, with full-length protein typically representing <50% of the total protein in the pooled fractions of Lip-$A3_{Axin}$, and <30% in those of Lip-$A5_{Axin}$. This explains the low (<1) molar ratios observed during ITC (see **Fig. 3** and accompanying text).

Gammons et al, Supplementary Fig. 3

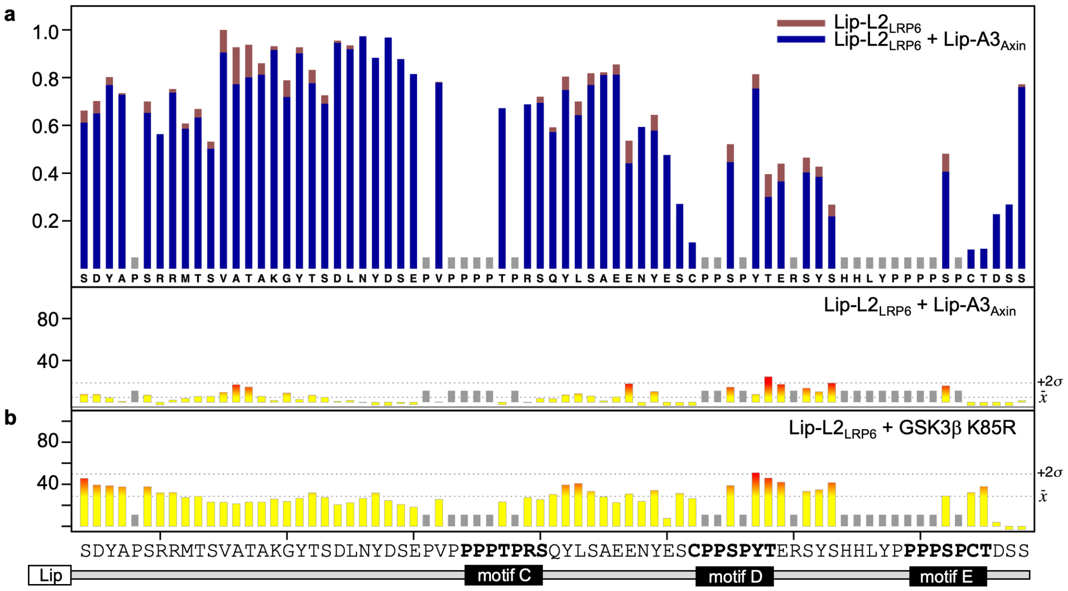

Supplementary Fig. 4. Interactions of Axin and GSK3 with the distal LRP6 ctail
(**a**) Relative peak heights in a BEST-TROSY spectrum of 80 μM ^{15}N-labeled Lip-L2$_{LRP6}$ incubated with 80 μM Lip-A3$_{Axin}$ and corresponding bleach map. (**b**) Bleach map generated from BEST-TROSY spectra of 80 μM ^{15}N-labeled Lip-L2$_{LRP6}$ incubated with 80 μM GSK3β-K85R. *Bottom*, sequence of distal L2 fragment of LRP6 spanning PPPSPxS motifs C-E; colors and graph labeling as in main **Fig. 2**. The bleach maps indicate very weak interactions of both proteins with the distal LRP6 ctail (around PPPSPxS motif D) that are however not measurable by ITC (see also main text).

Gammons et al, Supplementary Fig. 4

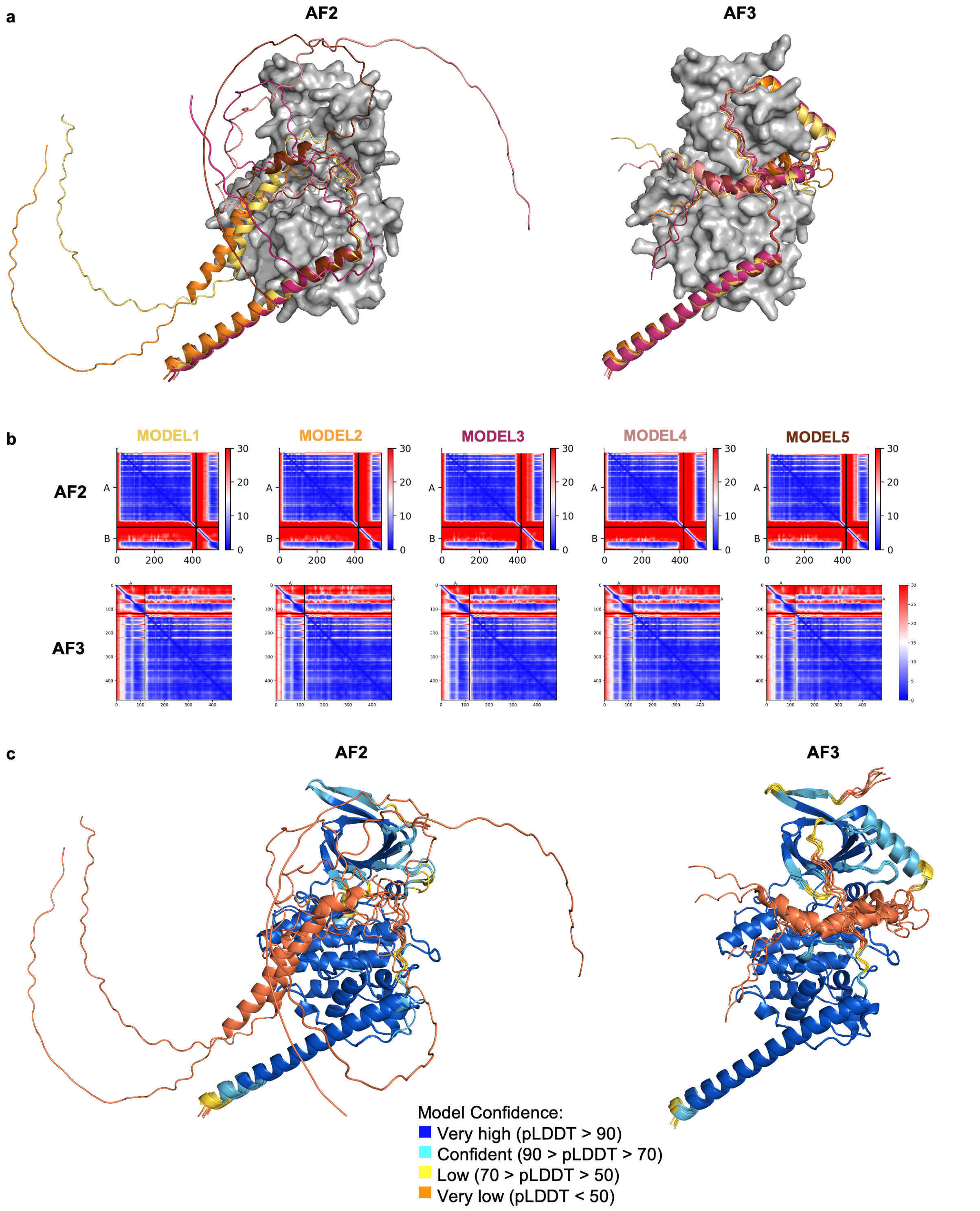

Supplementary Fig. 5. Models of Axin-GSK3 complexes predicted by Alphafold

(**a**) Predicted models of Axin-GSK3 complexes generated by AF2 (*left*) or AF3 (*right*); GSK3 is shown in a light grey surface representation, while the top five Axin models predicted by each Alphafold version are displayed in distinct colors, as indicated in the figure. (**b**) Predicted Aligned Error (PAE) plots for the top-5 models generated by AF2 or AF3; *blue*, regions of low error; *red*, regions of high error; colors in (**a**) correspond to those of top-5 models in (**b**). (**c**) Predicted Local Distance Difference (pLDDT) plots for the top-5 models generated by AF2 or AF3; colored according to confidence levels, as indicated in key.

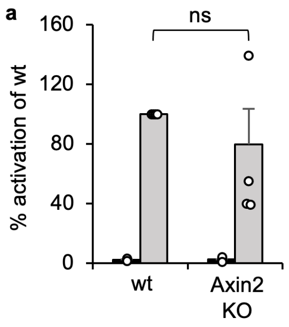

Supplementary Fig. 6. Testing Axin1 mutants in a complementation assay of Axin2 KO mutant HEK293T cells
(**a**) SuperTOP assays in wt and Axin2 KO HEK293T cells, showing that these Axin2 KO cells are fully Wnt-responsive; *circles*, values obtained from each independent experiment (n = 3); One-way ANOVA with multiple comparisons.

```
                                            ---------LIRup-----------------------
AXIN1_HUMAN          REFRYGSWREPVNPYYV-----NAGYALAPATSA-NDSEQQSLSS--D----ADTLSL-T      336
AXIN2_HUMAN          SGYRSFKRSDPVNPYHI-----GSGYVFAPATSA-NDSEISSDAL--T----DDSMSM-T      315
X.laevis             REFRPGTWREPVNPYYV-----NTGYAGAPVTSA-NDSEQQSMSS--D----ADTMSL-T      336
D.rerio              REYRHASWREPVNPYYV-----NSGYALAPATSA-NDSEQQSMSS--D----ADTLSL-T      339
C.intestina          DESHRQDVNRPAAPYYT-----ANTYW-VPPASA-IGSDQASKSS--DAT--SDTLSCPD      380
D.melanogaster       YVY-----NP------------STTNTSYVPNSR-VDSERASVSS--GGRTDSDTMSI-S      382
A.mellifera          IYL-----QHGTSPYHMLAPRLHAQYSSYNPVSR-QDSELQSLSS--DARTESDNMSL-T      342
P.dumerilii          EGLFLQPSKRKVNPYHI-----SY-----APTSA-QDSELQSLSS--DAL-TDDTISL-T      370
M.yessoensis         FPVPFAPQRSVAHPYHV-----SY-----APVSA-QDSEIQSLSS--DAL-TDDTRSI-D      369
T.spiralis           ---RGTRGGVPPNPYHT-------KYVPYLPTSA-QDSELQSMSS--DAA---------T      556
S.purpuratus         ---LYPGLLGGQNPYHP-----GC-AAIAPATSN-NDSELQSLSS--DAI-SDDTMSL-T      323
T.castaneum          LIY-----RSS---------GAHVAYNSYNPVSR-QDSEMQSLSSHSDARTESDNMSL-N      355
A.queenslandica      ------------LPHHT--------KSVYTPRNAHSDTEH--SSS---------------      290
H.vulgaris           ------------DIYHT------------------QAS----ESG-------ED------      228
Conservation

                     ------- ----------LIRα--------                          ----
AXIN1_HUMAN          DSSVDGIPPYRIR-----KQHRREMQESVQVN--GRVPLPHIPRTYRVPKE--V-RVEPQ      386
AXIN2_HUMAN          DSSVDGIPPYRVGS---KKQLQREMHRSVKAN--GQVSLPHFPRTHRLPKE--MTPVEPA      368
X.laevis             DSSVDGIPPYRLR-----KHYRREMQESANAN--GRGPLPHIPRTYHMPKD--I-HVDPE      386
D.rerio              DSSVDGVPPYRYR-----KPHRREIHESAKVN--GRVPLPHIPRTNRIPKD--I-HVEPE      389
C.intestina          NSSIDGATAYSSSRTGHKRYIRNQMKKN-PAN--VLPECCHQPRTTRCKDIPTLATNNPA      437
D.melanogaster       SCSMDGRPYIQRRHSSTESK---AIRQSAMANKET-NTFQVIPRTQRLHSNE-HRPLKEE      437
A.mellifera          DSSVDGASISKQRSKKQYIRQSRAIKDSASLNRDPLAHHTIIPRTQRVPRDL-MHPLKPE      401
P.dumerilii          DSSVDGRDSKRWN------KPHRKAVHRARLNHSAEVNSHFIPRTERAPKGCNLAETDPK      424
M.yessoensis         SSV---DNDKMWK------RQRRAMRKRAEQNRESGIHQQFIPRTQRAPKDSNIAEVNPK      420
T.spiralis           DSSFGNSR---SR-----HKEKRIIRQHIEQNRDN--------RTQ---REVVARDMMDE      597
S.purpuratus         DSSVDGFPVRSRQQQ--KRRQKRNMKYNIRQN--GKV-MTHLPCPRAHPRE--LPPTDPV      376
T.castaneum          ESNVDGRPGRSLRKSIEE-KQMRQF----ARNKEVNMNQRFIPRTQRMDVGN--NCLPIE      408
A.queenslandica      -------------------HPRRQQ---------YAGPTTHISRN---------KVLTQN      313
H.vulgaris           ------------------------FHH--HCNDYVPEMPTFQSRTQRMDS---MKQLAPE      259
Conservation

                     --GIR-----------
AXIN1_HUMAN          KFAEELIHRLEAVQRTREAEEKLEERLKRVRMEEEGEDGDPSSGPPG-------------      433
AXIN2_HUMAN          TFAAELISRLEKLKLELESRHSLEERLQQIREDEEREGSELTLNSRE-------------      415
X.laevis             KFAAELISRLEGVLRDREAEQKLEERLKRVRAEEEGDDGDVSSGPSV-------------      433
D.rerio              KFAAELISRLEGVLREREAQEKLEERLKRVRLEEEGDDADISTGPSL-------------      436
C.intestina          AFFEQLRIKLEKVLEEQKRVRAMMQRNNEP------------------------------      467
D.melanogaster       ELVSLLIPKLEEVKRKRDLEERARERNPGAALLTN------ERSS---------------      476
A.mellifera          EFAQVLIEKLENVKRERESQEKLDRHLQESDAINKDPSLEMSSGA---------------      446
P.dumerilii          AFADMLIERLERVIKDRGNLDKVKETMHSLDMTEEEEMKVGDVS----------------      468
M.yessoensis         KFAALLMEKLERVLKEREKEEKLHASMSRILESDLEEVDRSMLNRTVDRSVDRLERLDRS      480
T.spiralis           EFKFQVVEKLENLRRERETYEKLNESLQRLSEPENNSNSHRAA-----------------      640
S.purpuratus         EFAKKLTEKLEKVLQEREAKERLAAKLRKVEEEEHESEPPSST-----------------      419
T.castaneum          EHAALLIKKLENLKREQENQELFKRRLKEGDSLSS------LDDK---------------      447
A.queenslandica      QFYEIVCEKLSAVQRDRTAMKQRARNIARIAGKSYEDIMSIDWF----------------      357
H.vulgaris           QFAKLLTEKLERLLLDREAENFTDPQLLNQNVLSSH-------GLPI-------------      299
Conservation

                                                                -------BIR-------
AXIN1_HUMAN          -----PCHKLPPAPAWHH-------FPPRCVDMG-CAGL--RDAHEENPESILDEHVQRV      478
AXIN2_HUMAN          -----G------APT-QH-----------------PLSLLPSGSYEEDPQTILDDHLSRV      446
X.laevis             -----ISHKLPSGPPMHH-------FNSRYSETG-CVGMQIRDAHEENPESILDEHVQRV      480
D.rerio              -----ANHRVPPAVHVQH-------YGGRYSEMS-YNGLQLRDAHEENPESILDEHVQRV      483
C.intestina          ------------------------------------------NVFDEDIDEMMESHIARV      485
D.melanogaster       -------------------------ASDRAFA---EAIREKFALDEDNDQDILDQHVSRV      508
A.mellifera          -------------------------LSAKALA---DALREKLMIEEDNDHAILDQHVSRV      478
P.dumerilii          ----LAPTKGPNVS----------AIG----NLGALMATAMAEQDEDNAQSILEEHVSRV      510
M.yessoensis         IDRSIDRSMDRSIDRLDRSLDRSAMLRNTSSATSMPLMTSAVIMDDDNPNSILEDHVSRI      540
T.spiralis           -------------------------------VEKFCKVEKSFRAAPEDAEAILDDYYNRV      669
S.purpuratus         -------TMRPPG----------------------TQIFPVHDFVEDDPNSILEDHMSRV      450
T.castaneum          -------------------------FQTRDIE---SAMREKLQFQDDNDQDILDEHVSRV      479
A.queenslandica      ----------------------------------------------DTPEA-----IKYV      366
H.vulgaris           ----------------QR-----------------------------SYKPALSDVDSQS      314
Conservation

AXIN1_HUMAN          -LRTPGRQSPGP--GHRS------PDS-----GHVAK------------MPV--------      504
AXIN2_HUMAN          -LKTPGCQSPGV--GRYS------PRSRSPDHHHHHHSQYHSLL-----PPG--------      484
X.laevis             -MKTPGCQSPGT--GRHS------PKSRSPDGHLSKT------------LPG--------      511
D.rerio              -MKTPGCQSPGT--GRHS------PKSRSPDGLPAGK------------IPG--------      514
C.intestina          -IHSPGEHNIPP--ILSS------TRLPI------SA---------STSYP---------      512
D.melanogaster       -WKD---QTPHRSPGTMS------PCPPIPSRRRTATH----------------------      536
A.mellifera          -WSD---LTPSRSPGLSS------PRLHSPERRRSTHNY---------------------      507
P.dumerilii          -WDNSAGQTPSRSPGRSP------DR-R------RAAAQIMSAASSNISGPS--LLSRSL      554
M.yessoensis         -WESSAQQTPTRSPGQQS------PSLK------RSAADRKSSANPNQTMP----LPNTL      583
T.spiralis           GWKDSPKQSPMRSPGFAFGSRKKSPGSKSPDRRVISRMM-------TTSYPLPYAMSQTL      722
S.purpuratus         -FKDSNRHTP----RGFS------PRSRSPPDHHRSKL-----------PPS--------      480
T.castaneum          -FSD---HSLSISPGLVS------PKASSPPRGRW-------------------------      504
A.queenslandica      -CFDDEGTVAGADDGLHS------PPLSLPPPHKQSVGKRTTVES-SDTPPS--STSGAP      416
H.vulgaris           -WVMPPASVRYSVAGASS------TSGS-----CVNIEQ----------YP---------      343
Conservation

variable  conserved
```

Supplementary Fig. 7. Evolutionary conservation of Axin fragment spanning Lip-A3

Sequence alignment of Axin orthologs, indicating conservation of residues in key regulatory elements (LIRup, LIRα, PRTxR motif); GIR, GSK3 interacting region; BIR, β-catenin interacting region; S1-S4 serines are colored and underlined (see also main **Fig. 4**).

Gammons et al, Supplementary Fig. 7

```
LRP6_HUMAN                      ----VHGPASVPLGYVPHPSSLS-------GS-LPGM-SRGKSMISSLSIMGGSSG-PPY      1460
LRP5_HUMAN                      -----SGTPHVPLNFIAPGGSQH-------GP-FTGI-ACGKSMMSSVSLMGGRGGVPLY      1473
Xenopus.laevis                  ----VHGPVSVPLAYVPHPSSLT-------GS-LPGM-SRGKSVIGSLSIMAGSSG-PPY      1460
Danio.rerio                     ----VHGPPPVPLGYVPHPASLS-------GS-LPGM-SRGKSVMGSLSIMGGSSG-PPY      1464
Drosophila.melanogaster         --PS-----------TLSKSQR------------VSKIASVADAVRMSTLNSRNSMNSY      1528
Trichinella.spiralis            TTAEGSGARTVS-----TTVEYRYRL----------------GVGKPSIL-SRSVEPLH      1513
Anopheles.gambiae               --PG-----------GLNKNIR------------VSKLASVADAVRMSTLNSRTSGGSY      1457
Danaus.plexippus                --LALNKPATRPRTDAPSSDS----------------------FLRLSAMGTASGCGS-      1445
Nematostella.vectensis          VTPVLHGHASSLSSSSSSRASICAALPVRNGSTTSGSNKRQKPLPGPTSLSG-T-SQTPY      1492
Camponotus.floridanus           --PKPNNNRTVKPIFASQKNN-RKDVG-TGGGLKAGM------EAVRMSMLNGSSFGSSY      1532
Tribolium.castaneum             --PM-----------QPKPHIK-----------I--RKGIPDVVRMSMMT--GSETSY      1450
Strongylocentrotus.purpuratus   LMLNQLGKEHITHKYLNSGRP-H-------PK-TTNL-NSCSSKVSPKSGSGGESSTGFY      1486
Hydra.vulgaris                  ----------------------SEI----GSLTGESNYTKSSISTYVSTMSKNTDKSFY      1483
Conservation

                                                                   --------B element------
LRP6_HUMAN                      DRAHVTGASSSSSS----STKGTYFPAILNPPPSPATERSHYTMEFG------Y------      1504
LRP5_HUMAN                      DRNHVTGASSSSSS----STKATLYPPILNPPPSPATDPSLYNMDMF------Y------      1517
Xenopus.laevis                  DRAHVTGASSSSSS----STKGTYFPPILNPPPSPATERSHYTMEFG------Y------      1504
Danio.rerio                     DRAHVTGASSSSSS----STKGAYFPPILNPPPSPATVRSQYTMEFG------Y------      1508
Drosophila.melanogaster         DRNHITGASSSTTNG---SSMVAY---PINPPPSPATR---------------------      1560
Trichinella.spiralis            SRPDLTGKSSSSNSD--SGVKIGITLPPINPPPSPVSGTVVSTCSSDAQRIGKWLLQANR      1571
Anopheles.gambiae               DRNHITGASSSTTNG---SSLIGY---PLNPPPSPATTAGSTGR---------Y------      1496
Danaus.plexippus                -----GSGSGSGSGWGSDSLCGRYPRPTANPPPSPATASG------------------      1480
Nematostella.vectensis          DRNHLTGASSSSSAC---TVISAYPREPLNPPPSPATERSLFTVRSR-----GY------      1538
Camponotus.floridanus           DRSHITGASSSTRG----SSAGGYPQETLNPPPSPATIASSTRC----------------      1572
Tribolium.castaneum             DRNHITGASSSTN-E---SSLTCYPREPLNPPPSPATTRGSS-----------------      1488
Strongylocentrotus.purpuratus   ERNLPTGASSCSSEMFSSTGTSNYPKETLNPPPSPATERSQCTTEIY------Y------      1534
Hydra.vulgaris                  DRNTVTGASSVVS---------DYSHIPLNPPPSPTTDRGFSVISDA-PRTYDY--TSCD      1531
Conservation

                                -- B element ------------
LRP6_HUMAN                      --SSN-SPSTHRSYSYRPYSYRHFAPPTTPCSTDVCDSDYA--------PSRR-------      1546
LRP5_HUMAN                      --SSN-IPATAR--PYRPYIIRGMAPPTTPCSTDVCDSDYS--------ASRW-------      1557
Xenopus.laevis                  --SSN-SPSTHRSYSYRPYNYRHFAPPTTPCSTDVCDSDYT--------PSHR-------      1546
Danio.rerio                     --SSN-SPSTHRSYSYRPYSYRHFAPPTTPCSTDVCDSDYT--------PGRR-------      1550
Drosophila.melanogaster         -----------SRRPYRHYKIINQPPPPTPCSTDICDESDS--------NYTSKS-----      1596
Trichinella.spiralis            NASLFAVGEAAPFHPFSCYRGN-SPPPPYSASVEMSNLAAI----CKAKPKMIGSIAIA-      1625
Anopheles.gambiae               -CN-------SSYHPYKHYKIINQPPPPTPCSTDACDESDS--------NYTSKS-----      1535
Danaus.plexippus                ----------GRRRAYRNYRASNRPPPPTPASTDANESENE--------HARA----PPP      1518
Nematostella.vectensis          -CESVMTPSTCP--SHRYYRTPRMPPPPTPASTDAEGSVKQ--------PSRHCRRSH--      1585
Camponotus.floridanus           --S---SSNASRYKPYRHYRSINQPPPPTPCSTDVCDESDS--------N----------      1609
Tribolium.castaneum             -PS-------SRYRPYRHYRSINQPPPPTPCSTDVCDESDC--------NYPTRS-----      1527
Strongylocentrotus.purpuratus   --APVQNPSLSNFRPYRHHKLRYIPPPPTPCSTDICEDSEP--------YSMGGAAAAPP      1584
Hydra.vulgaris                  QCS---------C----QTGEEYDAPPPTLASLDELELYCPINKHLQANWYHLDSISNNS      1578
Conservation

LRP6_HUMAN                      -----MT--------------SVATAKG--------------------------------      1555
LRP5_HUMAN                      -------------------------KASK-------------------------------      1561
Xenopus.laevis                  -----LT--------------SAAAAKG--------------------------------      1555
Danio.rerio                     -----APHA-----------SSATAAKG--------------------------------      1562
Drosophila.melanogaster         -------NS-----------NNSNGGATKHSS-------------------------SS--      1612
Trichinella.spiralis            ESFPEMATK-----------STV-EKKEKHDDEKITEE-EEEEPEDLAIEQ----EEIDS      1668
Anopheles.gambiae               -------NR-----------SQRGGGGGGHGS------------------------GTTG      1553
Danaus.plexippus                SPAPLL------------------------------------------------------      1524
Nematostella.vectensis          ---------------------RCRPSRGTLSR------------------------YNGS      1600
Camponotus.floridanus           ------------------------------------------------------------      1609
Tribolium.castaneum             ------------------------------------------------------------      1527
Strongylocentrotus.purpuratus   VPHHKYTNI-----------SKSRRKKGKY--------------------------SHSN      1607
Hydra.vulgaris                  HQVHNNSNKRVRSRNRNIRNNTSNKINGSHISGKGVSKVDSLQRQWLCTEESDLDSSYER      1638
Conservation

LRP6_HUMAN                      --YTSDLNYDSEPVPPPPTPRSQYLSAEENY--ESCPPSPYTERSYSHHLYPPPPSPCTD      1611
LRP5_HUMAN                      --YYLDLNSDSDPYPPPPTPHSQYLSAED-----SCPPSPATERSYF-HLFPPPPSPCTD      1613
Xenopus.laevis                  --YISDLNYDSEPVPPPPTPRSQYLSAEENY--ESCPPSPYTERSYSHHLYPPPPSPCTD      1611
Danio.rerio                     --YASDLNYDSEPLPPPPTPRSQYLSAEENC--ESCPPSPYTERSYSHQLYPPPPSPCTD      1618
Drosophila.melanogaster         AAACLQYGYDSEPYPPPPTPRSHYHSDVRIVPESSCPPSPSSRSSTYFSPLPPPPSPVQS      1672
Trichinella.spiralis            NASQPLLSGSSNPVPPPPSPRSSGSVASSVI--RRCAVNKQ-RPYLTRSPRNPPPSPNEL      1725
Anopheles.gambiae               GGTSSSRCYDPEPYPPPPTPRSHYHSDIGGMLPESCPPSPSSRSSTYFSPLPPPPSPVPS      1613
Danaus.plexippus                -----------------------Y------------------------------------      1525
Nematostella.vectensis          VTESTDLAYESDLFAPPPTPNTNYLSEATHLSDQECPPSPTATERSFFQPYPPPPSPVTE      1660
Camponotus.floridanus           --YPARYRYESEPFPPPPTPRSVYHSDAA----ISCPPSPSSRSSTYFSPLPPPPSPVP-      1662
Tribolium.castaneum             -------RYDAGPFPPPPTPRSHCHS-------ESCPPSPSSRSSTYFNPLPPPPSPVAS      1573
Strongylocentrotus.purpuratus   IANPEAVNYDSEPYPPPPTPHSQYLS--DNF--DSCPPSPSTERSFF-NPYPPPPSPATD      1662
Hydra.vulgaris                  EGNYGDVNNRLLIFDPPPTPCTNYLSEEDRPSSDT-ETSVVAYGNSIRTHFAPPPSPVS-      1696
Conservation

LRP6_HUMAN                      S--S-----      1613
LRP5_HUMAN                      S--S-----      1615
Xenopus.laevis                  S--S-----      1613
Danio.rerio                     S--S-----      1620
Drosophila.melanogaster         P--SRGFT-      1678
Trichinella.spiralis            SDASSNIN-      1733
Anopheles.gambiae               P--SRGFS-      1619
Danaus.plexippus                ---------      1525
Nematostella.vectensis          T--PSLV--      1665
Camponotus.floridanus           ---------      1662
Tribolium.castaneum             P--PRGYDS      1580
Strongylocentrotus.purpuratus   S--T-----      1664
Hydra.vulgaris                  ---------      1696
Conservation

variable  conserved
```

Supplementary Fig. 8. Evolutionary conservation of LRP6 ctail
Sequence alignment of LRP5 and LRP6 orthologs from multiple species indicating conservation of residues (*purple*, strong; *cyan*, weak), revealing that the YxxY motif (*yellow*) is evolutionary conserved across the animal kingdom while the classical YxxΦ motif (*green*) is rare outside vertebrates (where some invertebrates contain a tandem YxxYxxY motif, possibly compensating for the missing YxxΦ motif).

Gammons et al, Supplementary Fig. 8

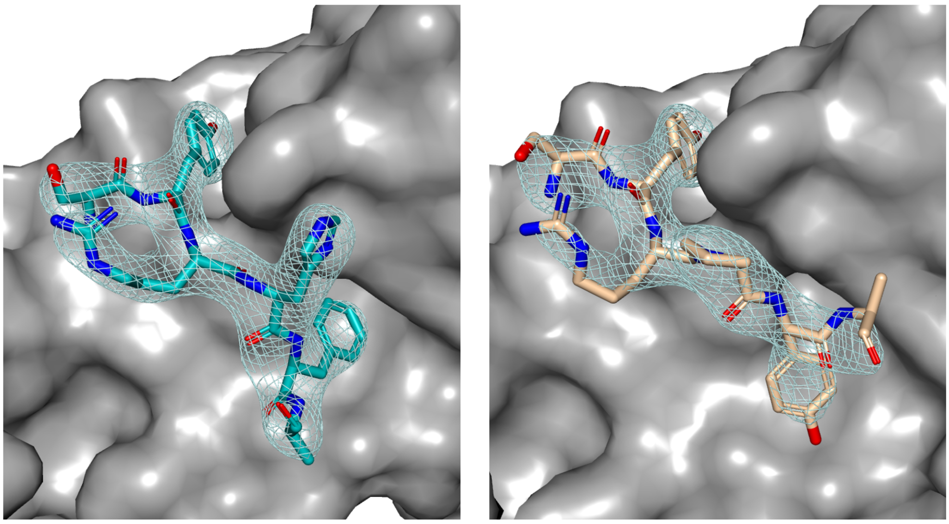

Supplementary Fig. 9. Contour maps corresponding to LRP6 YxxY and YxxF motifs bound to AP2μ
2Fc-Fo maps (contoured at 1.6σ) for YRHF (*teal*) and YRPY (*wheat*) in stick representation binding to the cargo-binding domain of AP2μ in surface representation (*gray*).

Gammons et al, Supplementary Fig. 9

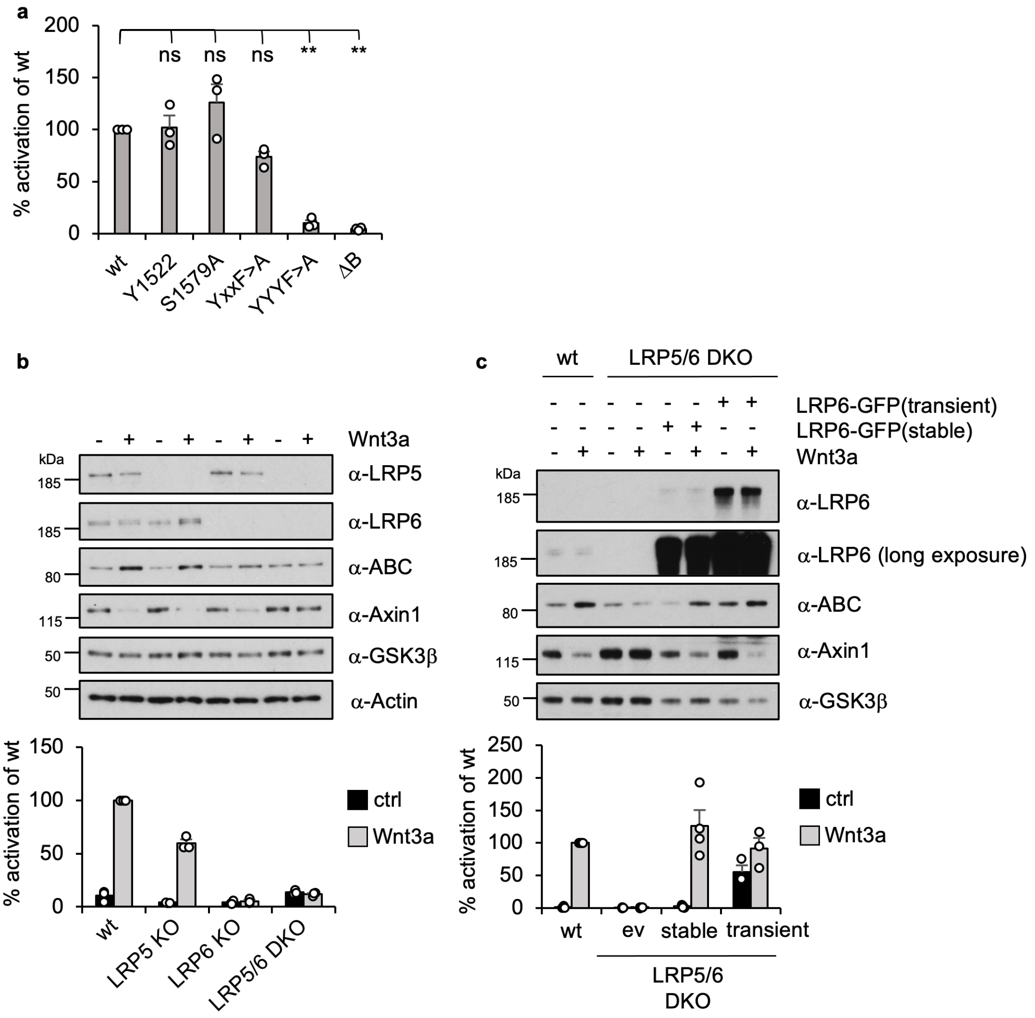

Supplementary Fig. 10. A complementation assay based on LRP5/6 DKO null mutant HEK293T cells

(**a**) SuperTOP assays in HEK293T cells transiently transfected with wt or mutant LRP6-GFP (as indicated); one-way ANOVA with repeated measures, statistical significance **, $p < 0.01$; *circles*, values obtained from each independent experiment (n = at least 3) in this and subsequent panels. (**b**) SuperTOP assays in wt and LRP5 KO, LRP6 KO and LRP5/6 DKO HEK293T cells +/- Wnt3a, and corresponding Western blots probed with antibodies as indicated on the right, revealing that LRP6 is critical for the Wnt response of these cells whereas LRP5 is dispensable. (**c**) SuperTOP assays in wt and LRP5/6 DKO HEK293T cells +/- Wnt3a, transiently (transient) or stably (stable) transfected with LRP6-GFP based on the pBABE vector (see Methods) and corresponding Western blots. In both cases, the Wnt response of the DKO cells is fully restored by the exogenous LRP6, however, this response is only Wnt-dependent in the stably transfected cells, likely because LRP6 is expressed at much lower levels in these cells than after transient transfection; positions of molecular weight markers indicated on the left (in kDa).

Gammons et al, Supplementary Fig. 10

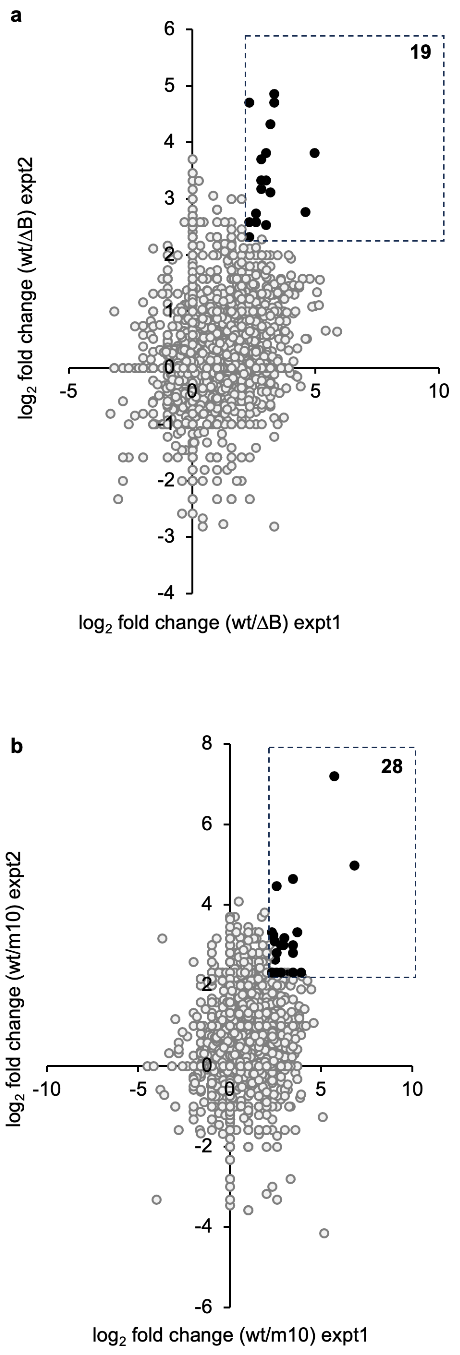

c

	expt1				expt2			
Protein	wt	wt+	ΔB+	m10+	wt	wt+	ΔB+	m10+
AAK1	0	7	1	4	3	9	1	11
ARHGAP32	42	103	11	33	103	112	84	56
CBLB	2	8	0	0	5	10	0	2
CEP135	3	8	0	0	37	56	4	46
CIT	23	31	0	2	6	14	0	3
FAM184A	0	5	0	3	27	26	0	18
FNDC3B	3	5	0	1	7	6	1	2
FUBP3	2	6	0	2	9	6	1	6
IOP7	1	7	0	1	12	10	1	13
KIAA0355	5	24	1	3	32	34	5	16
KIF7	9	15	3	9	4	5	1	5
NUP107	4	8	0	0	22	29	5	31
PIKFYVE	6	7	0	3	12	13	1	4
RAB3GAP1	1	10	1	3	31	29	1	7
SEC24D	3	6	1	10	19	20	3	13
SIPA1L1	3	9	0	2	8	20	1	3
SMCR8	0	7	0	1	13	10	1	3
TANC2	3	6	0	3	6	6	1	0
TRAPPC9	17	30	3	16	22	26	1	14
ANKRD50	4	17	3	3	18	25	16	4
AXIN2	2	5	1	0	5	5	4	0
CNP	2	11	3	2	19	17	16	2
DAAM1	4	11	4	1	27	25	7	1
DDX18	7	9	3	1	2	5	3	1
DYNC1H1	4	11	1	1	5	8	2	1
FAM50A	3	11	1	2	8	5	5	1
FRMD6	0	11	1	1	7	10	10	2
GPSM2	0	7	2	0	8	8	9	0
GSK3α	119	114	147	1	107	126	91	4
GSK3β	151	159	171	3	122	146	107	1
JPH3	5	4	4	0	2	5	2	1
KBTBD6	3	7	0	0	1	5	2	0
MOSPD2	0	7	2	0	17	20	14	4
NANP	3	15	5	1	6	10	4	2
PIN1	6	11	9	0	9	7	8	0
PLCB3	13	31	2	4	22	16	17	2
RPS6KA1	1	13	0	1	5	10	10	0
SEPT-3	2	5	2	1	8	5	10	1
SLC33A1	0	5	0	0	10	10	6	0
SNTA1	0	11	0	0	2	5	2	1
SNTB1	1	6	0	1	5	7	8	0
STON2	6	5	3	0	7	5	1	1
TTF2	2	6	1	1	22	22	7	1
TULP3	0	21	23	4	16	19	19	2
UBXN6	0	6	2	0	1	5	0	1
ZDHHC18	2	8	3	1	15	9	15	1

Supplementary Fig. 11. Identification of ΔB- and LRP6m10-sensitive TurboID hits
(**a, b**) Volcano plots showing additional (**a**) ΔB-sensitive or (**b**) LRP6m10-sensitive hits including those shown in main **Fig. 8** (black circles in dashed boxes), reduced >5x compared to wt in two independent TurboID experiments; *X axis*, expt1; *Y axis*, expt2; in cases where no peptide counts were identified, the 0 value was replaced with 1 for calculation of ratios. (**c**) Total unweighted spectral counts (>95% probability) of ΔB- or LRP6m10-sensitive hits (*shaded*) from the two experiments shown in (**a, b**) with (+) or without 2 hours of Wnt stimulation.

Gammons et al, Supplementary Fig. 11

kinase	kinase group	log_2 (score)	site percentile	percentile rank
GSK3B	CMGC	2.864	99.	1
GSK3A	CMGC	5.468	99.787	2
CDK8	CMGC	6.082	99.161	3
CDK19	CMGC	6.547	99.144	4
P38B	CMGC	6.789	99.096	5
KIS	Other	6.092	98.950	6
ERK5	CMGC	3.707	98.715	7
CDK7	CMGC	5.574	98.515	8
ERK1	CMGC	6.230	98.399	9
P38D	CMGC	6.413	98.070	10
CDK16	CMGC	5.968	97.944	11
P38G	CMGC	6.007	97.873	12
CDK18	CMGC	6.178	97.829	13
CDK17	CMGC	5.725	97.719	14
MPSK1	Other	2.378	97.604	15
MAK	CMGC	5.876	97.372	16
P38A	CMGC	5.297	97.133	17
JNK2	CMGC	5.290	96.714	18
CDK1	CMGC	5.248	96.471	19
ICK	CMGC	4.031	96.431	20
CLK3	CMGC	4.503	96.086	21
JNK1	CMGC	5.080	95.953	22
DYRK4	CMGC	5.250	95.936	23
HIPK2	CMGC	5.617	95.610	24
PASK	CAMK	1.787	95.424	25
DYRK1A	CMGC	4.238	95.063	26
ERK2	CMGC	4.079	94.979	27
HIPK4	CMGC	4.232	94.956	28
JNK3	CMGC	4.539	94.840	29
CDK3	CMGC	4.573	94.833	30
CDK13	CMGC	4.121	94.716	31
DYRK2	CMGC	4.580	94.711	32
CDK12	CMGC	4.138	94.010	33
DYRK1B	CMGC	4.360	93.824	34
MOK	CMGC	3.459	93.234	35
CDK14	CMGC	4.004	92.831	36
HIPK1	CMGC	4.157	92.426	37
CDK5	CMGC	3.909	91.986	38
GRK1	AGC	1.655	91.488	39
CK1D	CK1	0.584	90.720	40
CDK9	CMGC	3.315	90.664	41
CK1E	CK1	0.708	89.947	42
NDR2	AGC	1.269	89.561	43
CDKL5	CMGC	1.960	89.234	44
MASTL	AGC	-0.100	88.547	45
HIPK3	CMGC	2.995	87.251	46
PRKD1	CAMK	1.238	87.131	47
LATS1	AGC	1.072	87.005	48
CDK4	CMGC	2.852	86.668	49
NLK	CMGC	2.267	85.953	50
CHK1	CAMK	0.496	85.319	51
CDKL1	CMGC	1.373	84.383	52
ATR	PIKK	0.189	83.869	53
GRK7	AGC	1.459	83.822	54
CDK6	CMGC	2.418	83.621	55
DYRK3	CMGC	1.774	83.538	56
CDK10	CMGC	1.791	83.347	57
CLK2	CMGC	1.859	83.077	58
SRPK1	CMGC	1.197	80.057	59

Supplementary Fig. 12. Wnt-priming kinases for LRP6 S1490
List of kinases that can phosphorylate LRP6 S1490 based on Kinase Library (KL) scores >80.

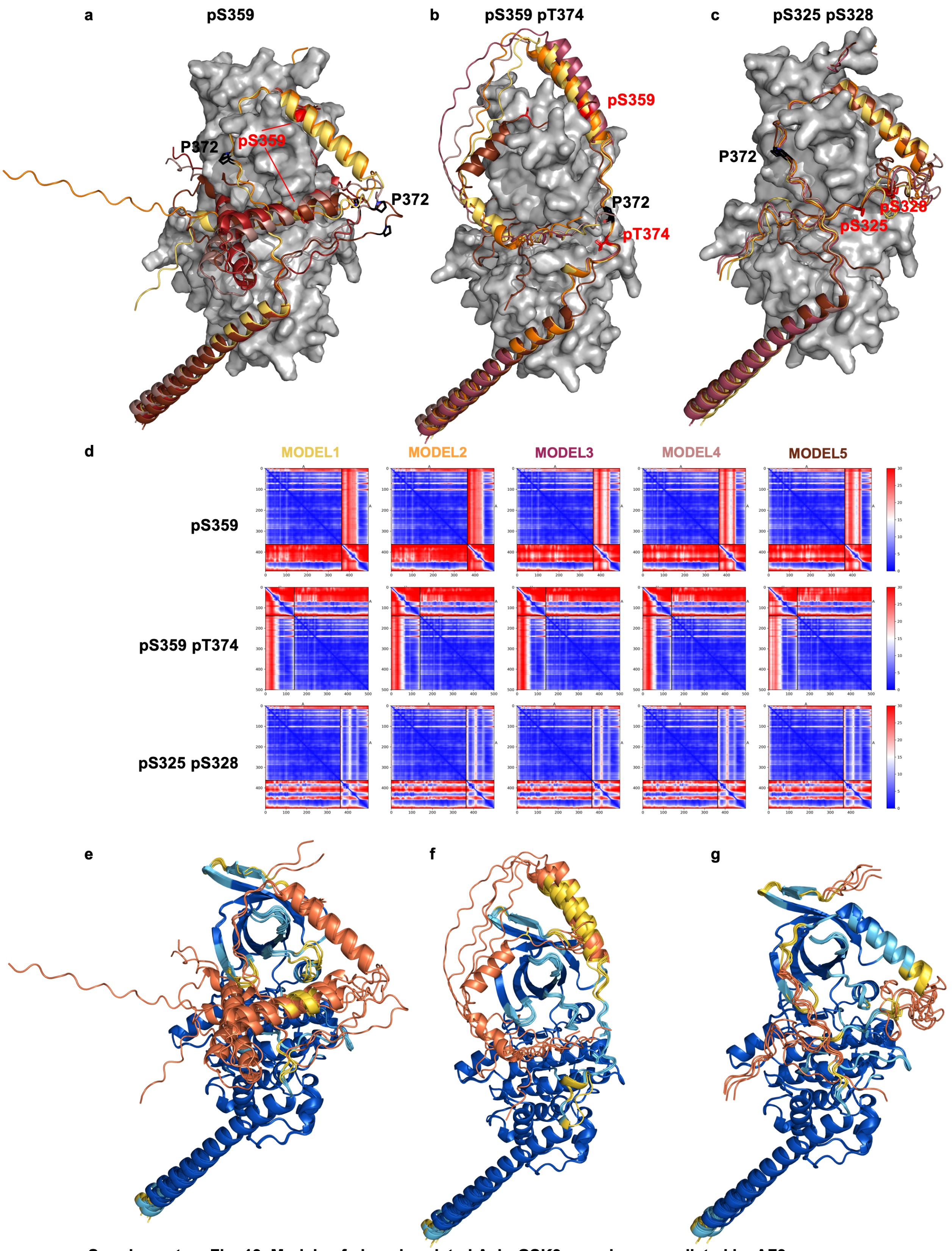

Supplementary Fig. 13. Models of phosphorylated Axin-GSK3 complexes predicted by AF3
(**a-c**) AF3 models of GSK3 binding to Axin phosphorylated at distinct sites as indicated above models; GSK3 is shown in a light surface representation, while the top-5 Axin models are displayed in distinct colors; *red*, phosphorylated residues; *black*, P372. (**d**) Predicted Aligned Error (PAE) plots for the top-5 models; colors in (**a-c**) correspond to the top-5 models in (**d**); *blue*, regions of low error error; *red*, regions of high error. (**e-g**) Predicted Local Distance Difference (pLDDT) plots for the models in (**a-c**); coloring reflects model confidence (pLDDT) as indicated in **Supplementary Fig. 5** (*dark blue*, >90; *light blue*, >70; *yellow*, >50; *orange*, <50).

Gammons et al, Supplementary Fig. 13

PDB ID	9FIW	9FIY
**Data collection**		
**Space group**	$P\,6_4$	$P\,6_4$
**Cell dimensions**		
***a, b, c* (Å)**	127.6, 127.6, 75.1	128.4, 128.4, 74.6
α, β, γ (°)	90, 90, 120	90, 90, 120
**Resolution (Å)**	48.6 – 2.81 (2.97 – 2.81)	48.7 – 2.88 (3.04 – 2.88)
R_{merge}	0.070 (1.58)	0.086 (1.63)
$I / \sigma I$	30.1 (2.2)	23.8 (1.9)
**Completeness (%)**	99.9 (99.5)	99.9 (99.6)
**Redundancy**	20.3 (20.7)	19.8 (20.1)
**Refinement**		
**Resolution (Å)**	48.6 – 2.81	48.7 – 2.88
**No. reflections**	16168	15176
R_{work} / R_{free}	0.192/0.246	0.191/0.242
**No. atoms**		
**Protein**	2068	2070
**Ligand/ion**	0	0
**Water**	0	0
***B*-factors**		
**Protein**	95.5	100.6
**Ligand/ion**	n/a	n/a
**Water**	n/a	n/a
**R.m.s. deviations**		
**Bond lengths (Å)**	0.008	0.007
**Bond angles (°)**	1.90	1.83

Supplementary Table 1. Crystal data collection and refinement statistics
Structures were collected and solved from a single crystal, values in parentheses are for highest-resolution shell.
