# Supplementary material for: Wnt signalosome assembly is governed by conformational flexibility of Axin and by the AP2 clathrin adaptor
Source: Nat Commun. 2025 May 21;16:4718. doi: 10.1038/s41467-025-59984-9 (PMC12095580; doi:10.1038/s41467-025-59984-9)
Supplement: Supplementary file 3 — Reporting Summary [file 41467_2025_59984_MOESM3_ESM.pdf]

nature portfolio

Corresponding author(s): Mariann Bienz

Last updated by author(s): 30/04/25

Reporting Summary

Nature Portfolio wishes to improve the reproducibility of the work that we publish. This form provides structure for consistency and transparency in reporting. For further information on Nature Portfolio policies, see our Editorial Policies and the Editorial Policy Checklist.

Statistics

For all statistical analyses, confirm that the following items are present in the figure legend, table legend, main text, or Methods section.

n/a	Confirmed	
☐	☒	The exact sample size (*n*) for each experimental group/condition, given as a discrete number and unit of measurement
☐	☒	A statement on whether measurements were taken from distinct samples or whether the same sample was measured repeatedly
☐	☒	The statistical test(s) used AND whether they are one- or two-sided<br>*Only common tests should be described solely by name; describe more complex techniques in the Methods section.*
☒	☐	A description of all covariates tested
☐	☒	A description of any assumptions or corrections, such as tests of normality and adjustment for multiple comparisons
☐	☒	A full description of the statistical parameters including central tendency (e.g. means) or other basic estimates (e.g. regression coefficient) AND variation (e.g. standard deviation) or associated estimates of uncertainty (e.g. confidence intervals)
☐	☒	For null hypothesis testing, the test statistic (e.g. *F*, *t*, *r*) with confidence intervals, effect sizes, degrees of freedom and *P* value noted<br>*Give P values as exact values whenever suitable.*
☒	☐	For Bayesian analysis, information on the choice of priors and Markov chain Monte Carlo settings
☒	☐	For hierarchical and complex designs, identification of the appropriate level for tests and full reporting of outcomes
☒	☐	Estimates of effect sizes (e.g. Cohen's *d*, Pearson's *r*), indicating how they were calculated

Our web collection on statistics for biologists contains articles on many of the points above.

Software and code

Policy information about availability of computer code

Data collection	n/a
Data analysis	n/a

For manuscripts utilizing custom algorithms or software that are central to the research but not yet described in published literature, software must be made available to editors and reviewers. We strongly encourage code deposition in a community repository (e.g. GitHub). See the Nature Portfolio guidelines for submitting code & software for further information.

Data

Policy information about availability of data

All manuscripts must include a data availability statement. This statement should provide the following information, where applicable:

- Accession codes, unique identifiers, or web links for publicly available datasets
- A description of any restrictions on data availability
- For clinical datasets or third party data, please ensure that the statement adheres to our policy

3D structures are available (https://rcsb.org, 9FIW; 9FIY). NMR assignments are available (https://bmrb.io/ #52482; #52483). Proteomics data will be released when the manuscript is accepted but can be accessed using the following details (Username: reviewer_pxd054245@ebi.ac.uk, Password: HU4gmPknRB2D).

Research involving human participants, their data, or biological material

Policy information about studies with human participants or human data. See also policy information about sex, gender (identity/presentation), and sexual orientation and race, ethnicity and racism.

Reporting on sex and gender	n/a
Reporting on race, ethnicity, or other socially relevant groupings	n/a
Population characteristics	n/a
Recruitment	n/a
Ethics oversight	n/a

Note that full information on the approval of the study protocol must also be provided in the manuscript.

Field-specific reporting

Please select the one below that is the best fit for your research. If you are not sure, read the appropriate sections before making your selection.

[x] Life sciences [ ] Behavioural & social sciences [ ] Ecological, evolutionary & environmental sciences

For a reference copy of the document with all sections, see nature.com/documents/nr-reporting-summary-flat.pdf

Life sciences study design

All studies must disclose on these points even when the disclosure is negative.

Sample size	No sample-size calculations were performed. All experiments (except for the TurboID screens) were repeated in at least triplicate.
Data exclusions	No data exclusions were applied. All experiments were run alongside positive and negative controls which were used to determine whether experimental treatments were successful.
Replication	As above
Randomization	No randomization was performed. As this is a molecular biology study, we ensured reproducibility through biological replicates, standardized controls and hypothesis-driven experimental design, rather than randomization.
Blinding	No blinding was performed, but the key experiments were performed independently by two team members. This is a mechanistic study that is based on rigorous experimental controls and standardized protocols, which generates objective and quantifiable data that are not influenced by researcher expectations.

Reporting for specific materials, systems and methods

We require information from authors about some types of materials, experimental systems and methods used in many studies. Here, indicate whether each material, system or method listed is relevant to your study. If you are not sure if a list item applies to your research, read the appropriate section before selecting a response.

Materials & experimental systems

n/a	Involved in the study
[ ]	[x] Antibodies
[ ]	[x] Eukaryotic cell lines
[x]	[ ] Palaeontology and archaeology
[x]	[ ] Animals and other organisms
[x]	[ ] Clinical data
[x]	[ ] Dual use research of concern
[x]	[ ] Plants

Methods

n/a	Involved in the study
[x]	[ ] ChIP-seq
[x]	[ ] Flow cytometry
[x]	[ ] MRI-based neuroimaging

Antibodies

Antibodies used	a-GFP (rabbit) Sigma Cat#G1544;<br>RRID: AB_439690

a-LRP5 (rabbit) Cell Signalling Technologies Cat#5731
RRID: AB_10705602
a-FLAG (rabbit) Sigma Cat#F7425;
RRID: AB_439687
a-HA (rabbit) Abcam Cat#ab9110;
RRID: AB_307019
a-active-b-catenin (ABC; rabbit) Cell Signaling Technologies Cat#8814S;
RRID: AB_11127203
a-GSK3b (rabbit) Cell Signaling Technologies Cat#9315;
RRID: AB_490890
a-LRP6 (rabbit) Cell Signaling Technologies Cat#2560;
RRID: AB_10831525
a-Axin1 (rabbit) Cell Signaling Technologies Cat#2087;
RRID: AB_2274550
a-actin (rabbit) Abcam Cat#ab8227;
RRID: AB_2305186

Validation

Antibodies against Axin1, GSK3-beta and LRP5/6 were validated by CRISPR/Cas9 deletion in HEK293T cells (Supplementary Figs. 6 & 10, and unpublished). FLAG, HA and GFP antibodies are routinely used in the lab and have been validated through a lack of signal in untransfected controls. Anti-active beta-catenin is an antibody widely used in the field that has been validated through its known specificity for Wnt-dependent stabilization of beta-catenin.

Eukaryotic cell lines

Policy information about cell lines and Sex and Gender in Research

Cell line source(s)	HEK293T (ATCC, CRL3216); Wnt3a L-cells (ATCC, CRL2647); L-cells (ATCC, CRL2648); TREx-293 (Invitrogen, R710-07)
Authentication	HEK293T were authenticated by STR profiling.
Mycoplasma contamination	All cells were routinely tested for mycoplasma (by PCR). A low dose of plasmocin was added to cells during CRISPR gene editing to protect against mycoplasma infection.
Commonly misidentified lines (See ICLAC register)	n/a

Plants

Seed stocks	n/a
Novel plant genotypes	n/a
Authentication	n/a
