# Supplementary material for: Wnt signalosome assembly is governed by conformational flexibility of Axin and by the AP2 clathrin adaptor
Source: Nat Commun. 2025 May 21;16:4718. doi: 10.1038/s41467-025-59984-9 (PMC12095580; doi:10.1038/s41467-025-59984-9)
Supplement: Supplementary file 4 — Source Data [file 41467_2025_59984_MOESM4_ESM.zip › Bienz Source Data/Bienz Source Data_blots.pdf]

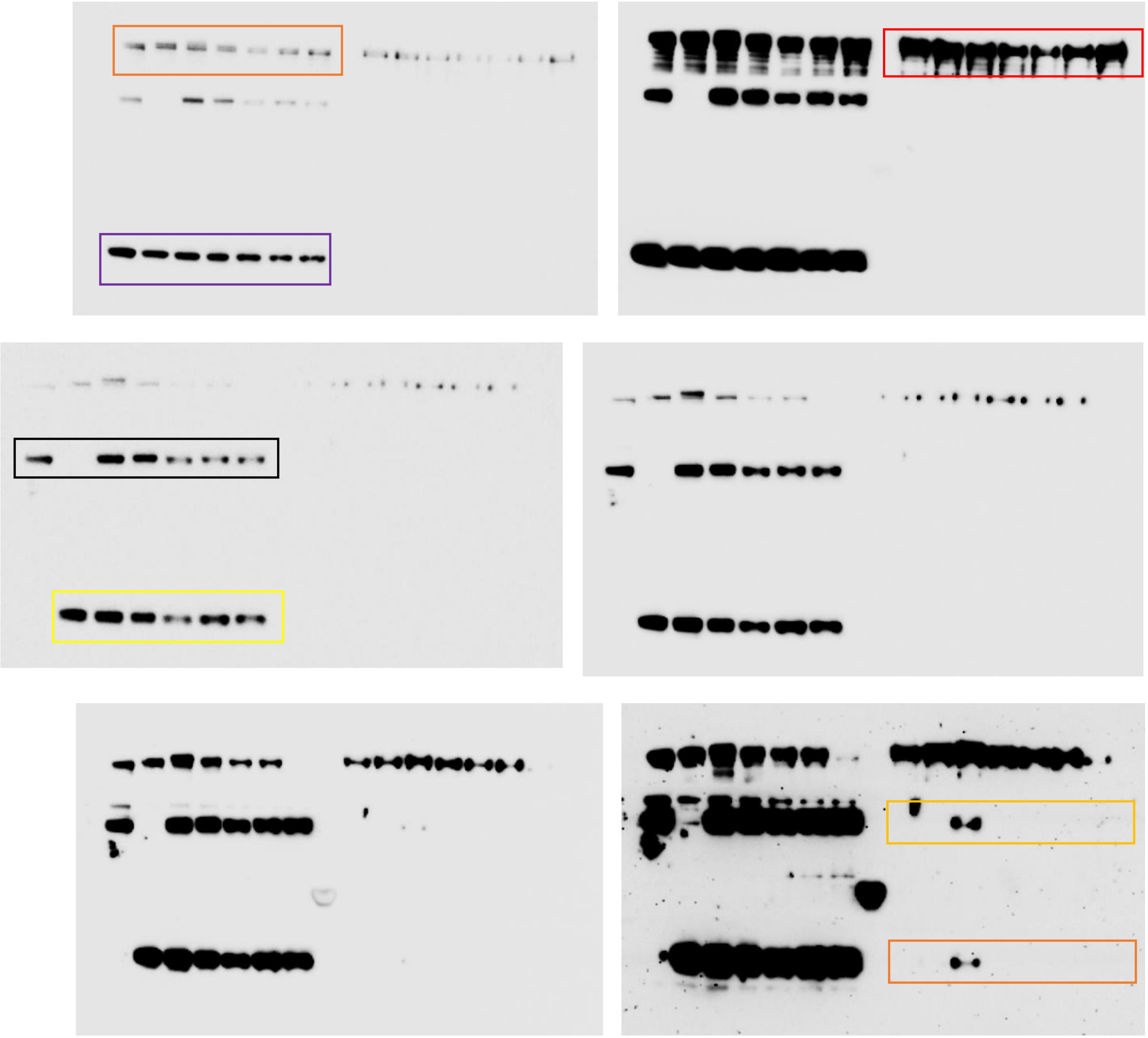

Blot 1

Blot 2

Blot 2

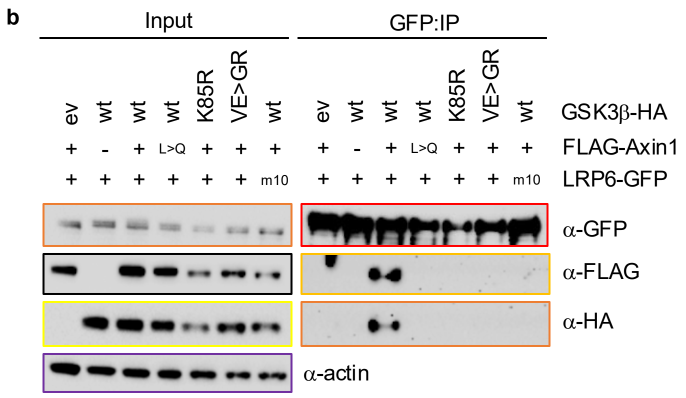

Following construct expression validation, samples were run on duplicate blots, which were cut and probed as follows:

Blot1 probed for LRP6 (anti-GFP, ~200kDa), Axin1 (anti-FLAG, ~115kDa) and Actin (~42kDa) – not lower exposures shown, Axin coIP visible as per blot 2 in longer exposures.

Blot2 probed for pS1490 LRP6 (not shown in figure, ~200kDa), Axin1 (anti-FLAG, ~115kDa) and GSK3 (anti-HA, ~50kDa)

Gammons et al, Figure 1b

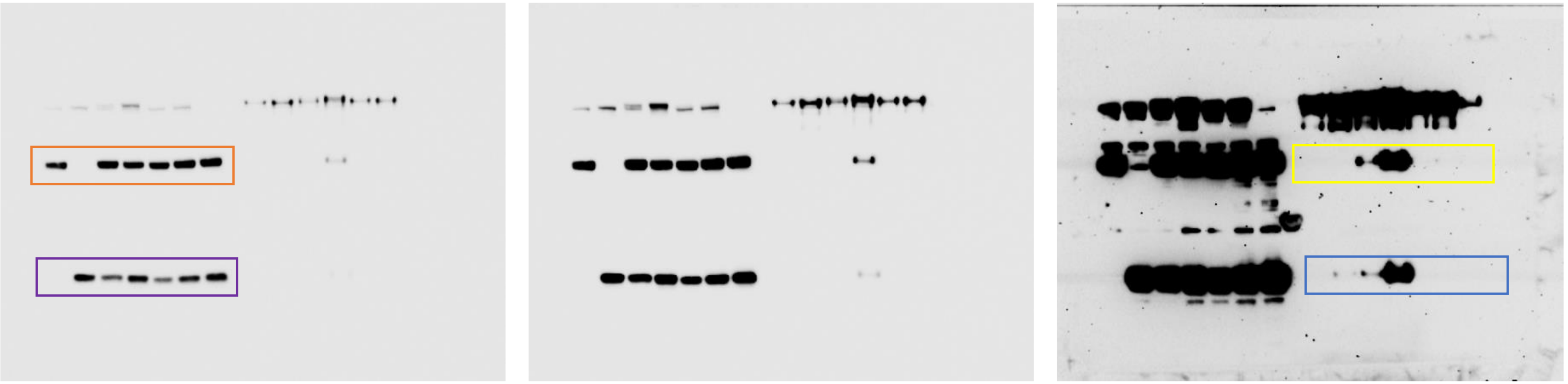

Blot 1

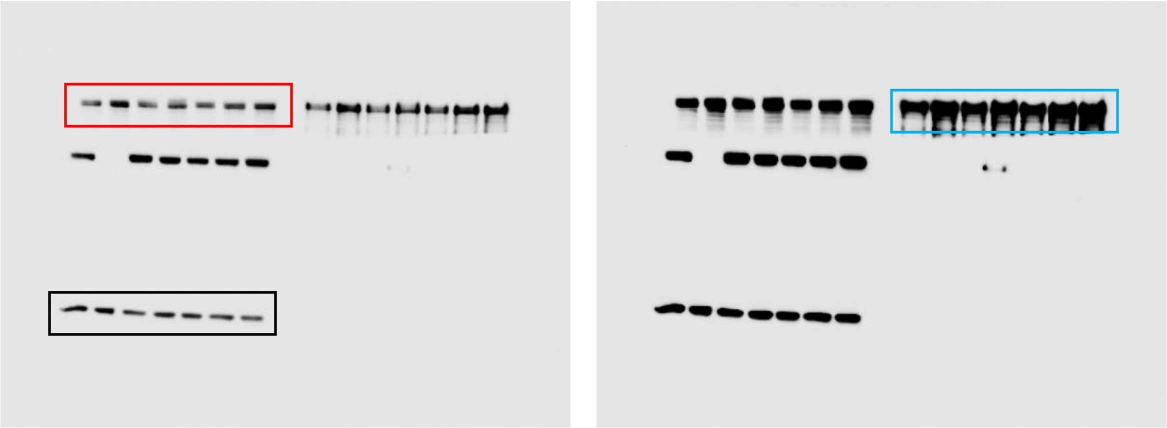

Blot 2

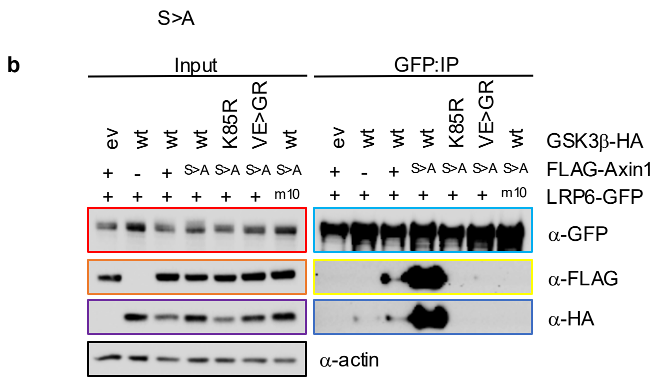

Following construct expression validation, samples were run on duplicate blots, which were cut and probed as follows:

Blot1 probed for pS1490 LRP6 (not shown in figure, ~200kDa), Axin1 (anti-FLAG, ~115kDa) and GSK3 (anti-HA, ~50kDa)
Blot2 probed for LRP6 (anti-GFP, ~200kDa), Axin1 (anti-FLAG, ~115kDa) and Actin (~42kDa)

Gammons et al, Figure 4b

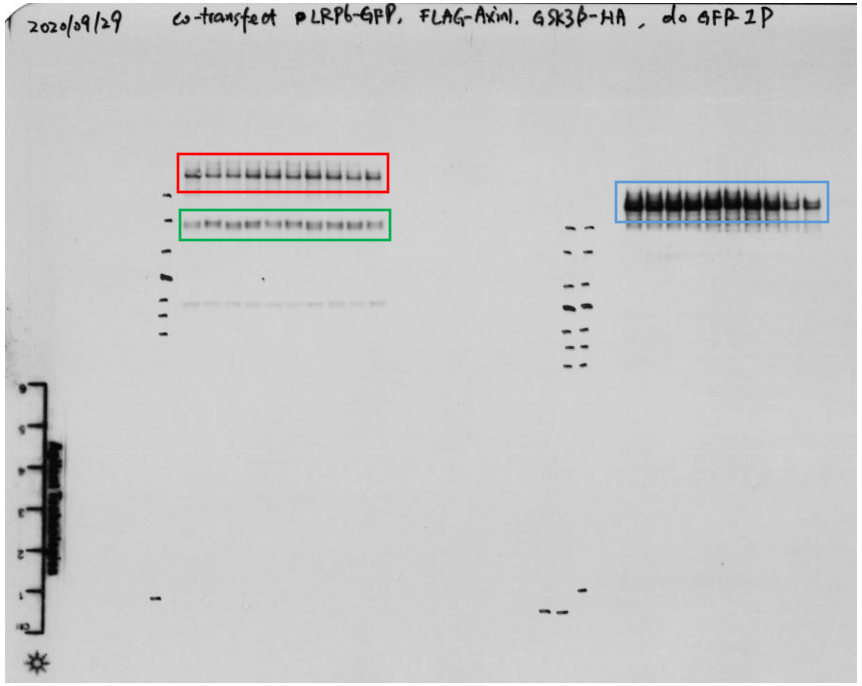

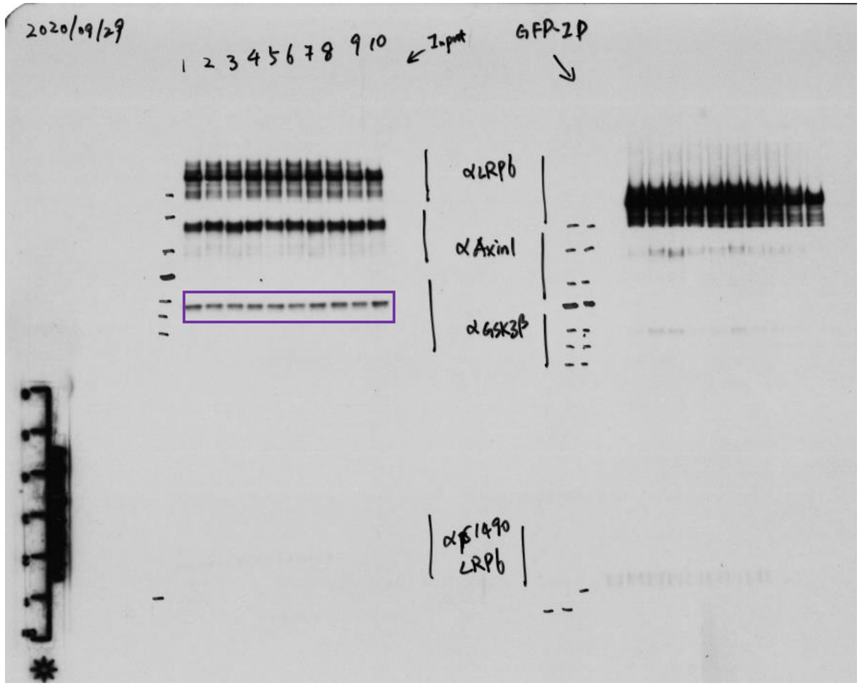

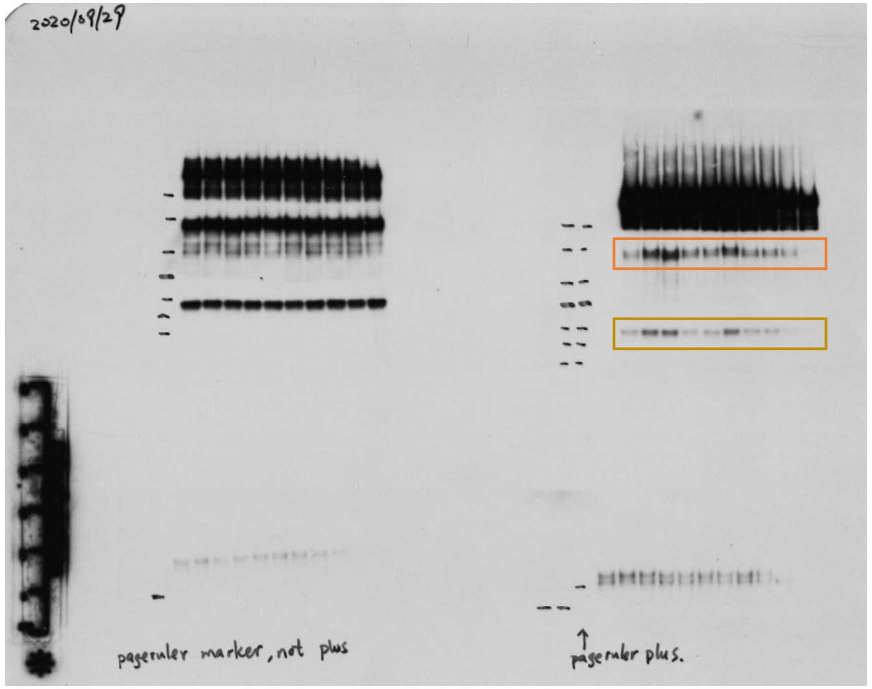

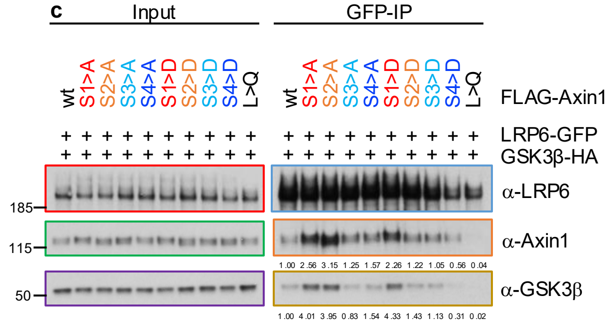

Following construct expression validation, samples were run on blots, which were cut and probed as follows:

Blot1 inputs samples, Blot2 GFP-IP samples.

Both blots probed for LRP6 (~200kDa), Axin1 (~115kDa) and GSK3 (~50kDa)

Gammons et al, Figure 4c

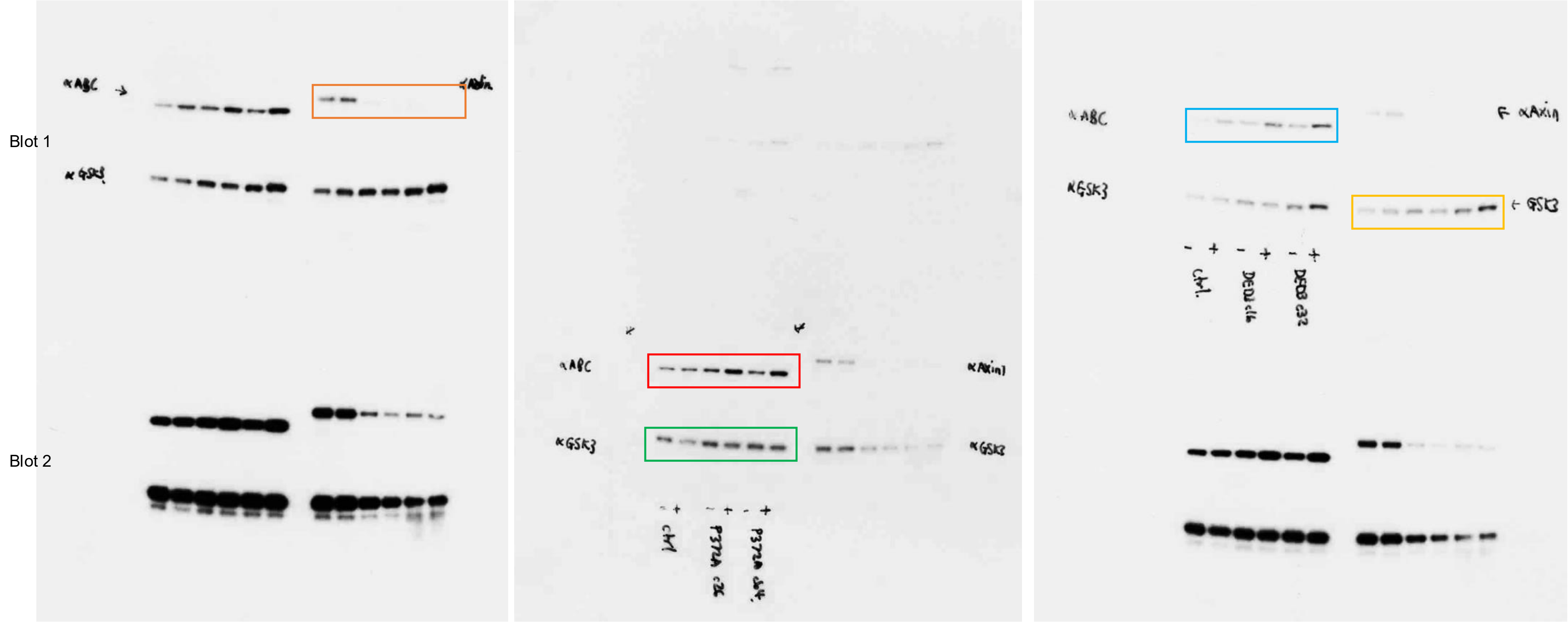

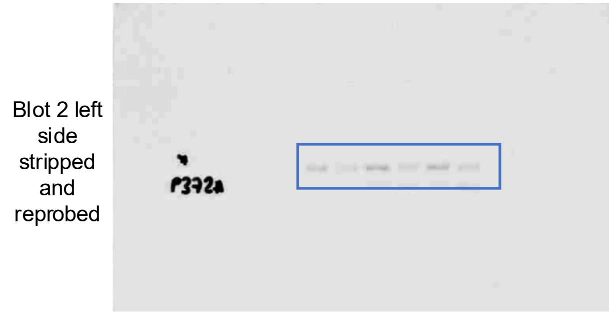

Blot 2 left side stripped and reprobed

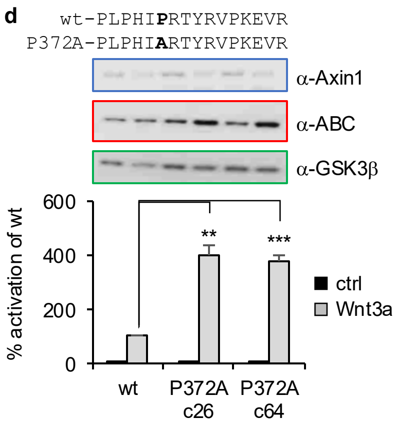

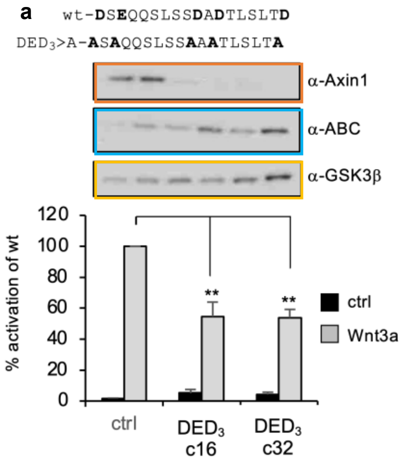

Following antibody validation, samples were run in duplicate on individual blots, which were cut and probed as follows:

Blot1 (top) control and DED3 samples (fig 6a) loaded in duplicate either side of a ladder cut horizontal (at 65kDa MW) and vertical through the ladder. Probed for anti-ABC (top, left blot), anti-Axin1 (top, right blot) and anti-GSK3 (bottom left and right)

Blot 2 (bottom) control and P372A samples (fig 6c) loaded only on to the left side of the blot. Left side cut at ~65kDa and top half probed for anti-ABC (as above) and bottom half probed for GSK3 (as above).

Blot2 was stripped and re-probed for anti-Axin1 (lower panel)

Gammons et al, Figure 6a,d

Blot 1

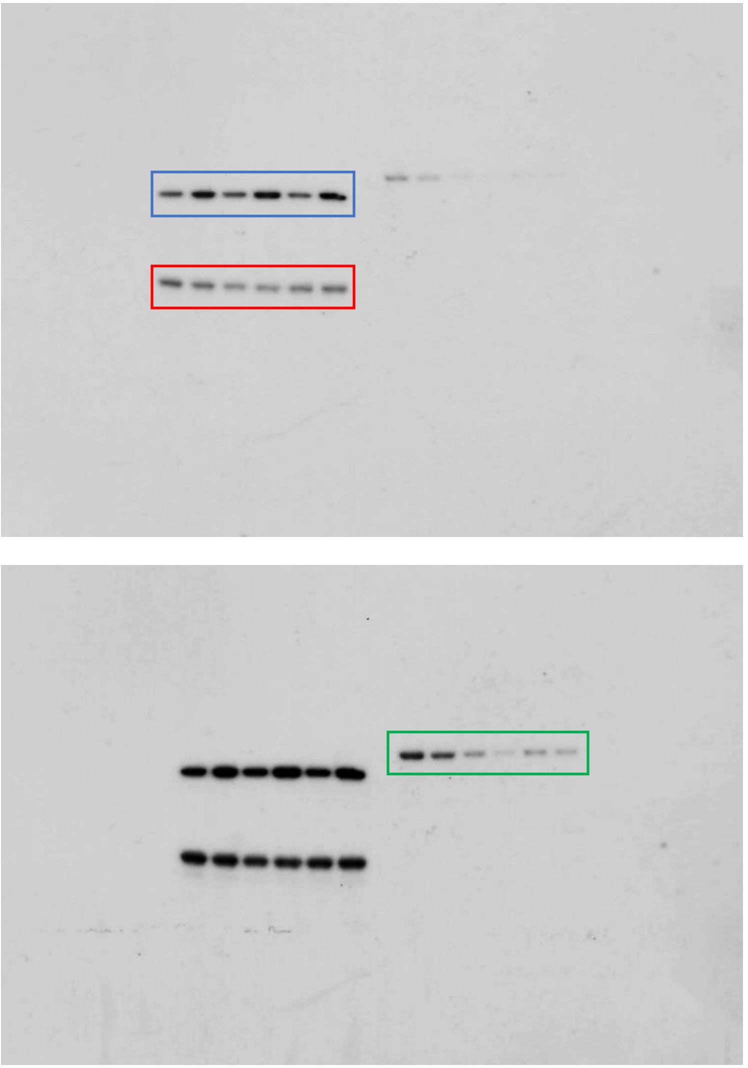

Blot 2

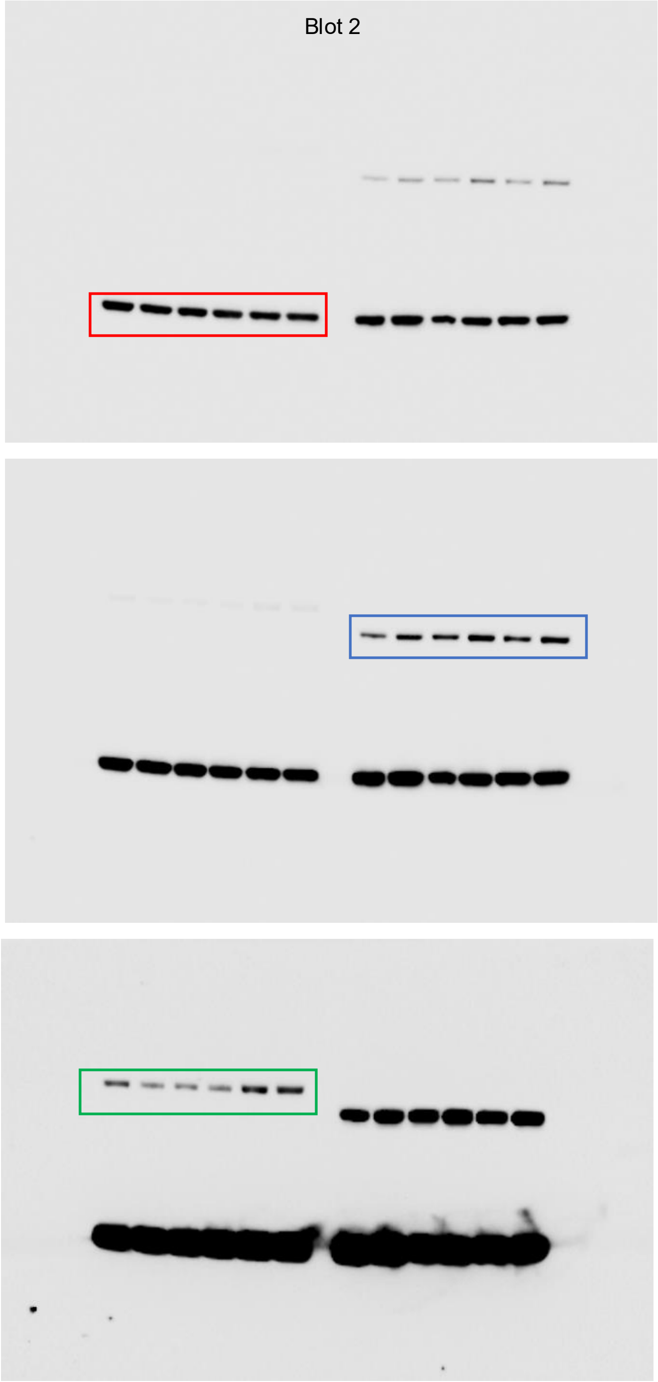

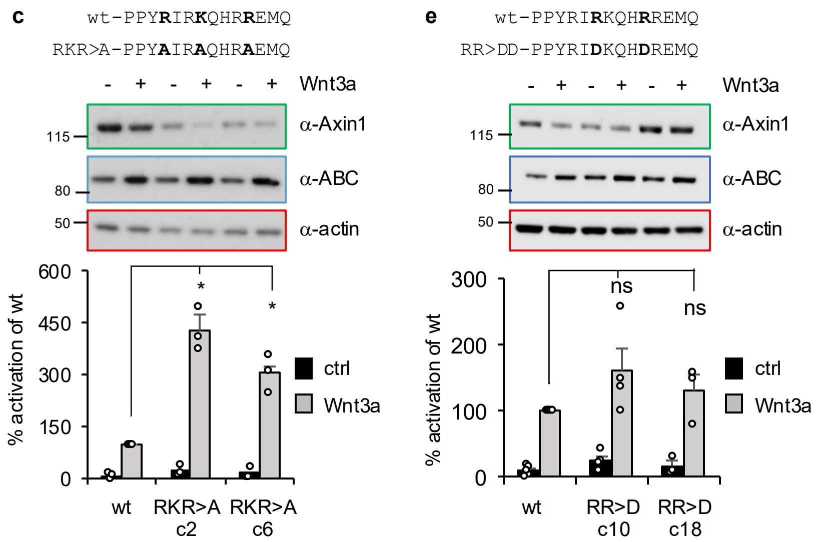

Following antibody validation, samples were run in duplicate on individual blots, which were cut and probed as follows:

Blot1 (left): control and RKR>A samples (fig 6d) loaded in duplicate either side of a ladder cut horizontal (at 65kDa MW) and vertical through the ladder.
Probed for anti-ABC (top, left blot), anti-Axin1 (top, right blot) and anti-GSK3 (bottom left)

Blot2 (right): control and RR>DD samples (fig 6e) loaded in duplicate either side of a ladder cut horizontal (at 65kDa MW) and vertical through the ladder.
Probed for anti-Axin1 (top, left blot), anti-ABC (top, right blot) and anti-GSK3 (bottom left and right)

Gammons et al, Figure 6c,e

Unfortunately, the unprocessed & uncropped scan for the blot shown in Fig. 6b was lost during a lab move. However, duplicate samples shown in this blot (for mutant line c28) are included in the blot shown in **Fig. 6f** (to compare the mutant lines shown in Figs. 6a-e) for which we have provided an unprocessed scan of the original uncropped blot.

e

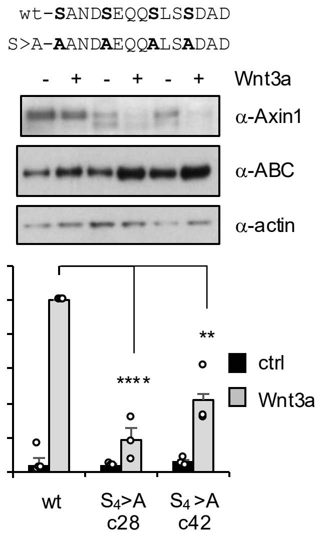

Gammons et al, Figure 6b

Blot 1

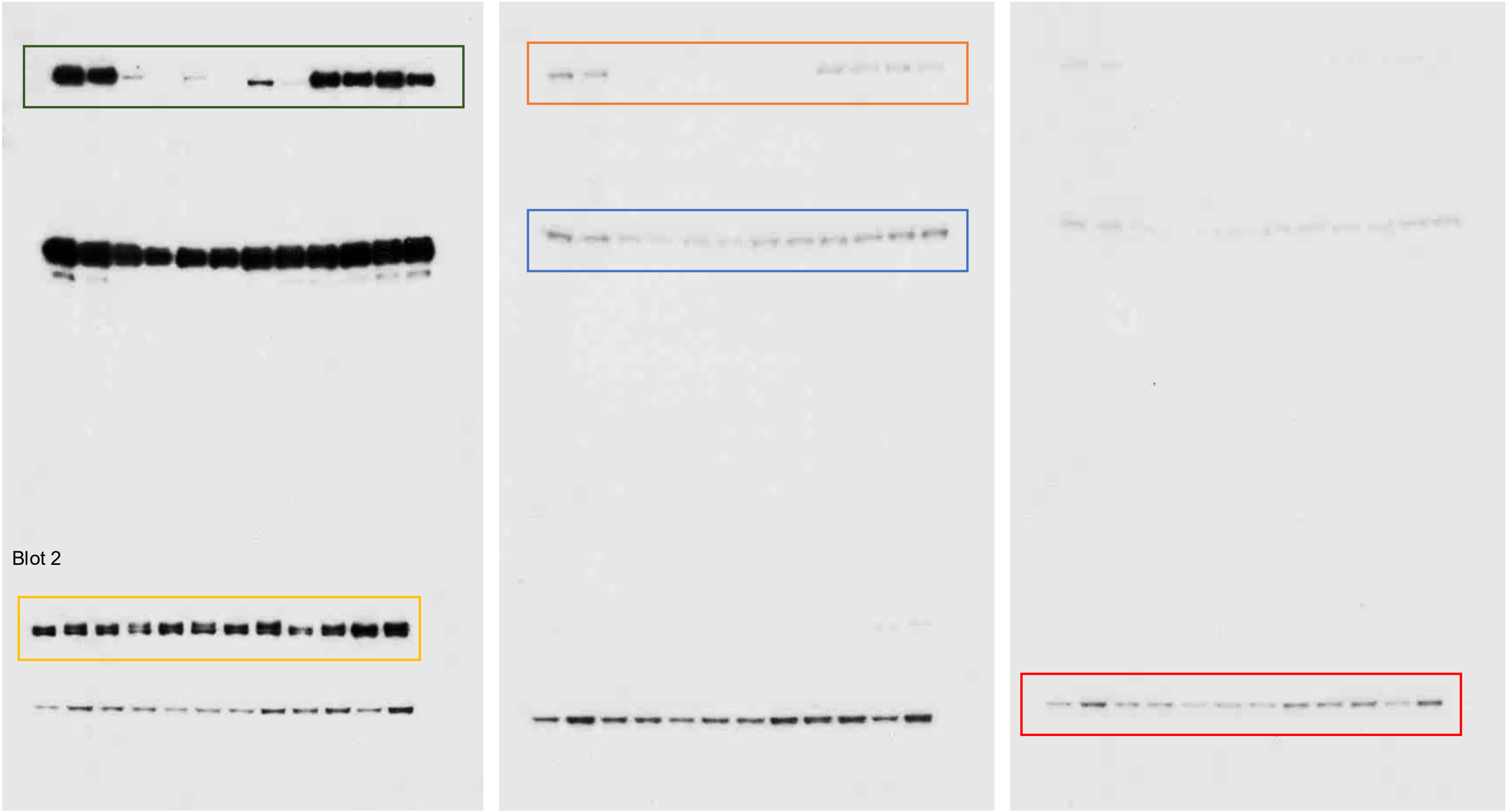

Blot 2

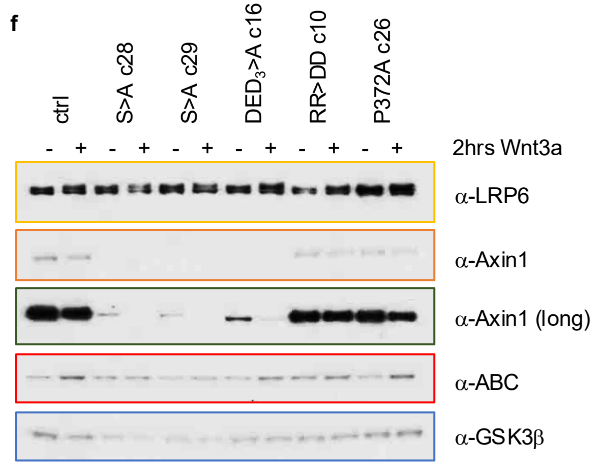

Following antibody validation, samples were loaded on duplicate blots, which were cut and probed as follows:

Blot1 (top): cut horizontal (at 65kDa MW) and probed for anti-Axin1 (top, ~115kDa), and anti-GSK3 (bottom, ~46kDa)

Blot2 (bottom): cut horizontal (at 65kDa MW) and probed for anti-LRP6 (top, ~200kDa), anti-ABC (bottom, ~80kDa).

Gammons et al, Figure 6f

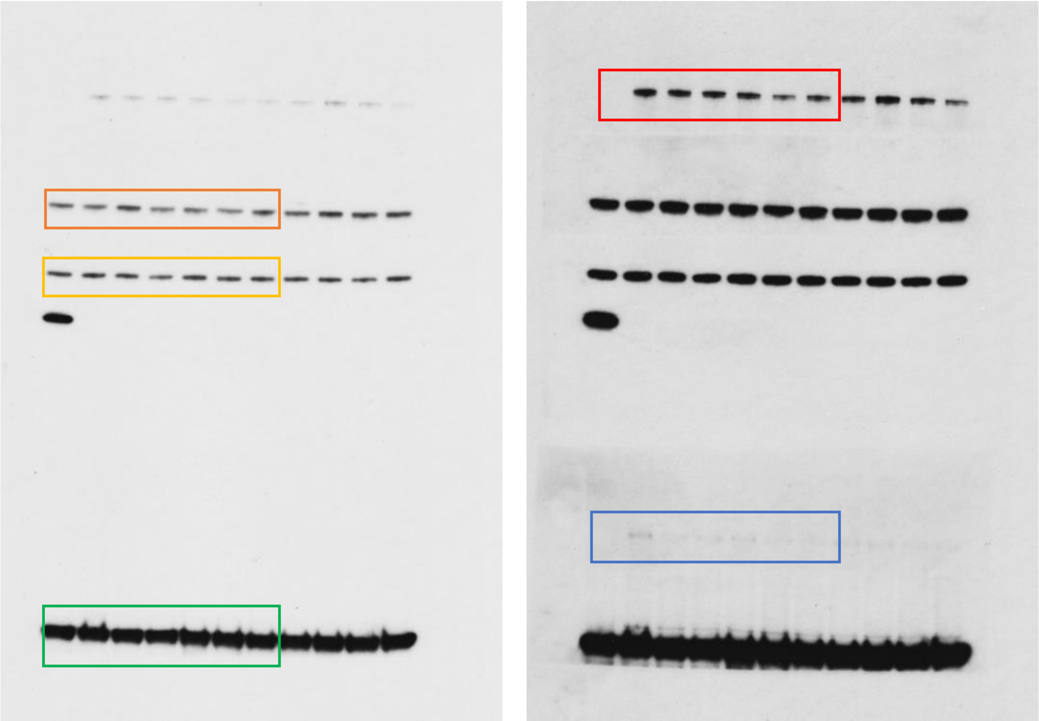

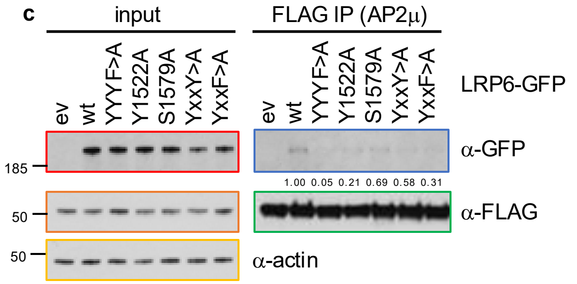

Following construct expression validation, samples were loaded on two blots and loaded with either input (blot 1) or IP (blot 2) samples. Only samples 1-7 are shown in the final figure.

Blot1 (top): cut horizontal (at 140kDa MW) and probed for anti-GFP (top, ~200kDa), and combined anti-actin and anti-FLAG (bottom, ~42kDa and ~50kDa). Note in lane one a band at ~25kDa is seen corresponding to the FLAG-GFP empty vector transfected negative control.

Blot2 (bottom): cut horizontal (at 40kDa MW) and probed for anti-FLAG and anti-GFP (top, ~50kDa and ~200kDa).

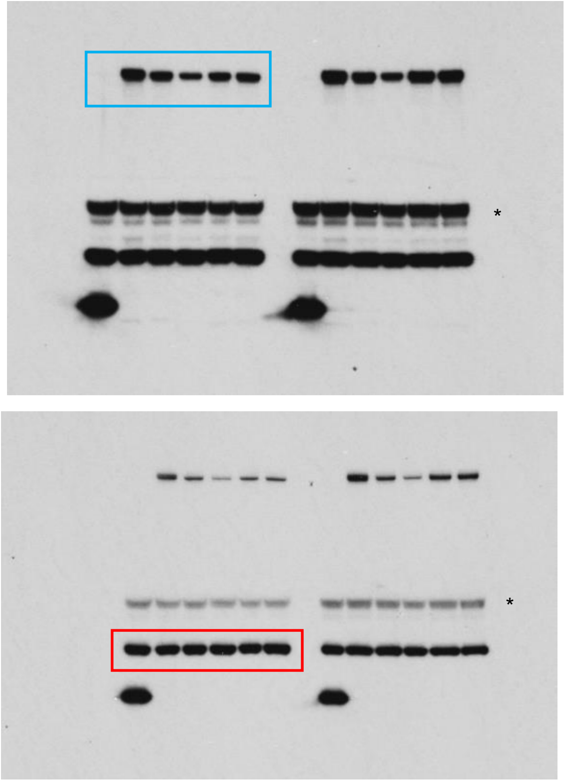

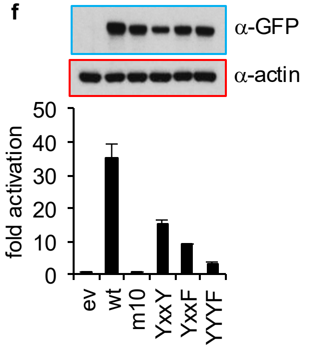

Samples loaded in duplicate separated by a ladder. No cutting performed, blot probed with a mix of anti-GFP and anti-actin. Non-specific band observe, as indication (asterix)

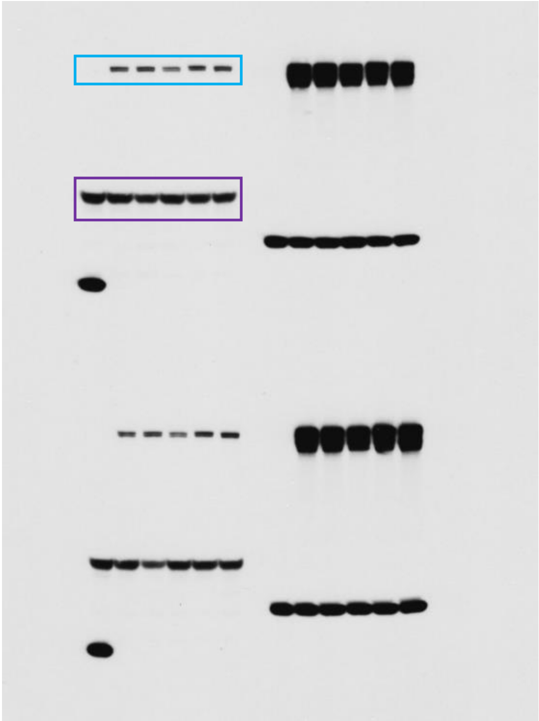

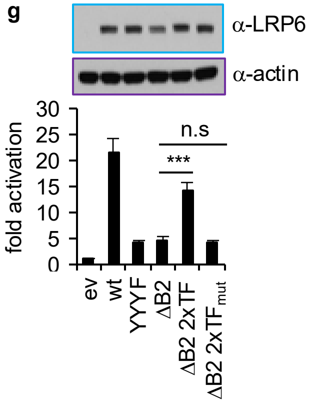

Samples loaded ileft hand side of ladder. Blot cut at ~140kDa and ~30kDa; probed for anti-LRP6 (top), anti-actin (middle) and anti-GFP (bottom).

Gammons et al, Figure 7c,f-g

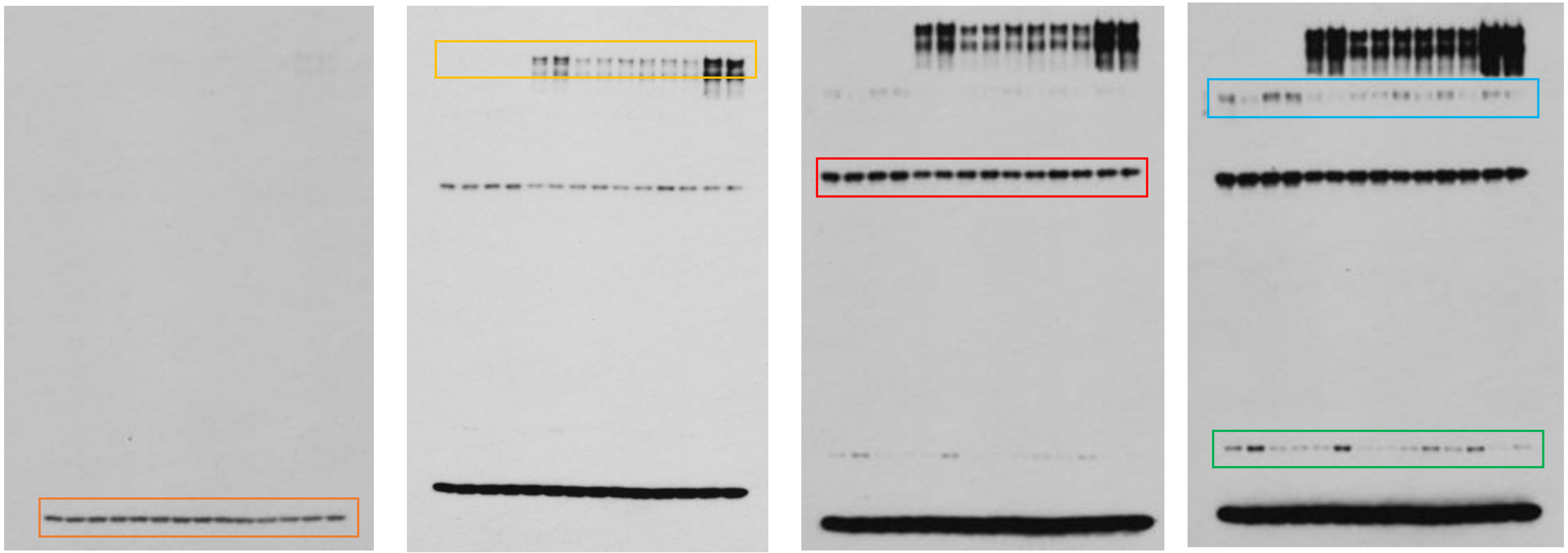

h

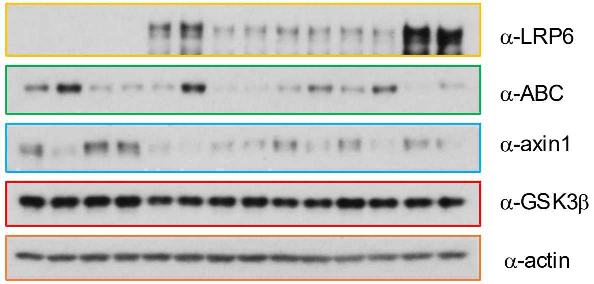

Following construct expression validation, samples were loaded on two blots in duplicate.

Blot1 (top): cut horizontal (at 140kDa and 60kDa MW) and probed for anti-LRP6 (top, ~200kDa), anti-Axin1 (middle, ~115kDa), and anti-GSK3b (bottom, ~46kDa).

Blot2 (bottom): cut horizontal (at 60kDa MW) and probed for anti-ABC (top, 85kDa) and anti-Actin (bottom, 42kDa).

Gammons et al, Figure 7h

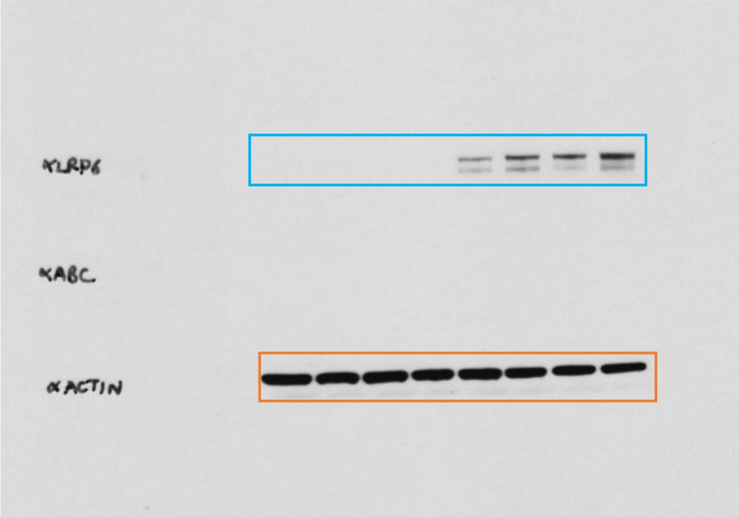

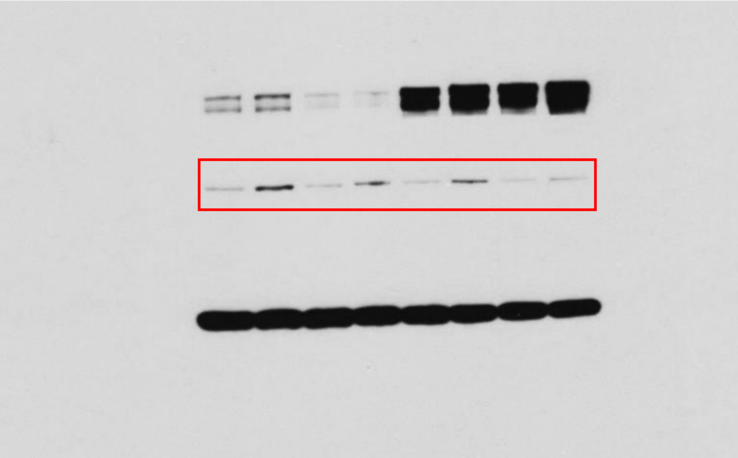

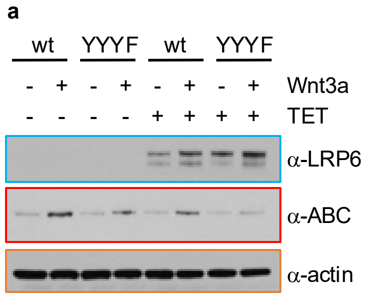

Following construct expression validation, samples were run on a single blot. Two exposures are shown.

Blot1: cut at 140kDa and 60kDa and probed for LRP6 (top, anti-LRP6, ~200kDa), ABC (middle, anti-ABC) and anti-actin (bottom, 42kDa).

Gammons et al, Figure 8a

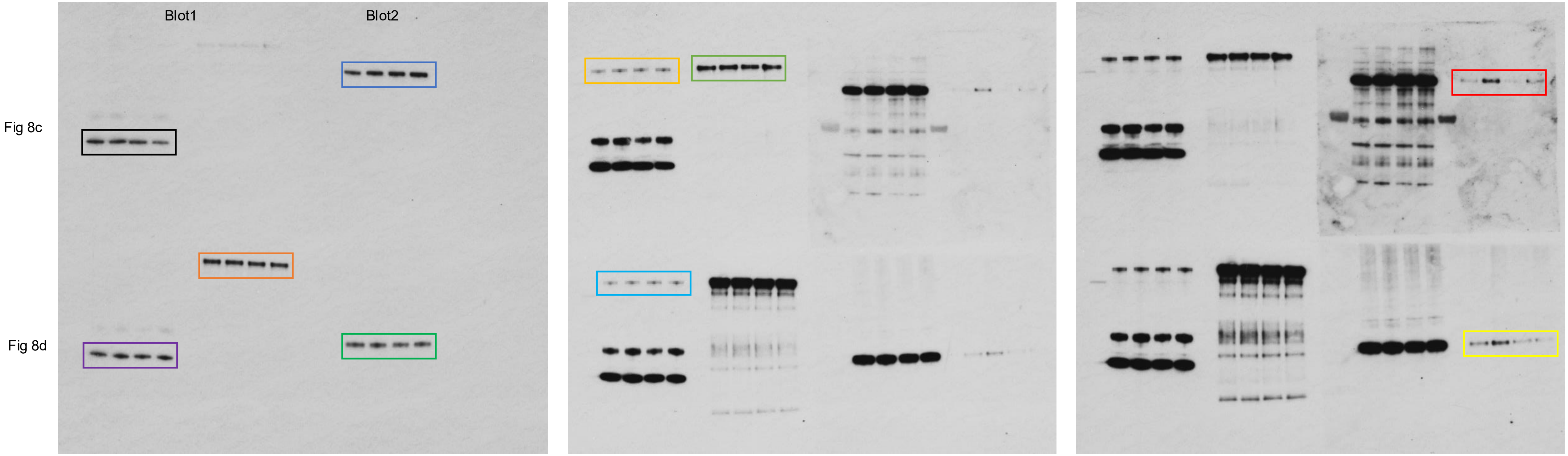

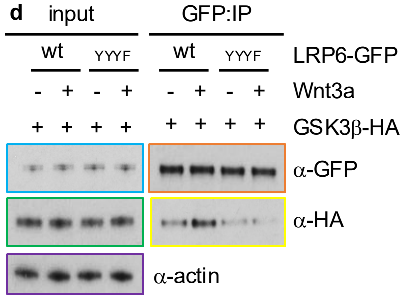

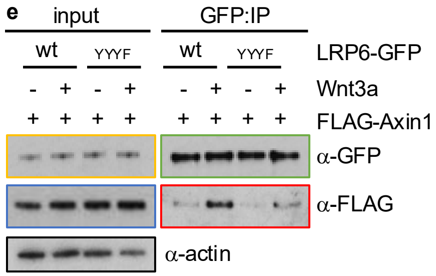

Following construct expression validation, samples were run on duplicate blots, each loaded input (left) and IP (right). Blots for fig 8c are shown bottom and fig8d, above.

Blot1's: cut at 140kDa and probed for LRP6 (top, anti-GFP, ~200kDa), and anti-actin (bottom, 42kDa).

Blot2's: top (fig8d) probed for Axin1 (anti-FLAG, ~115kDa), and bottom (fig8c) for GSK3b (anti-HA, ~50kDa).

Gammons et al, Figure 8d-e

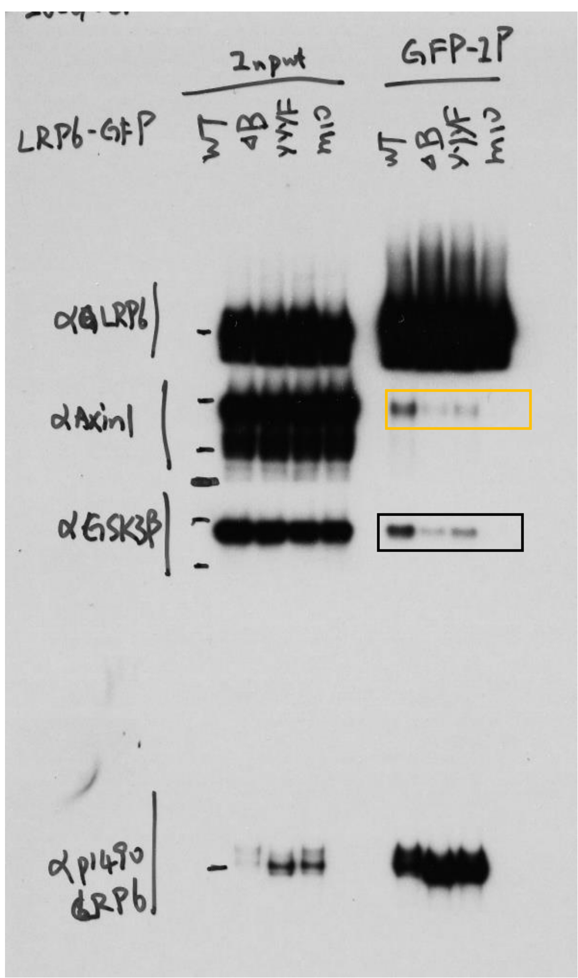

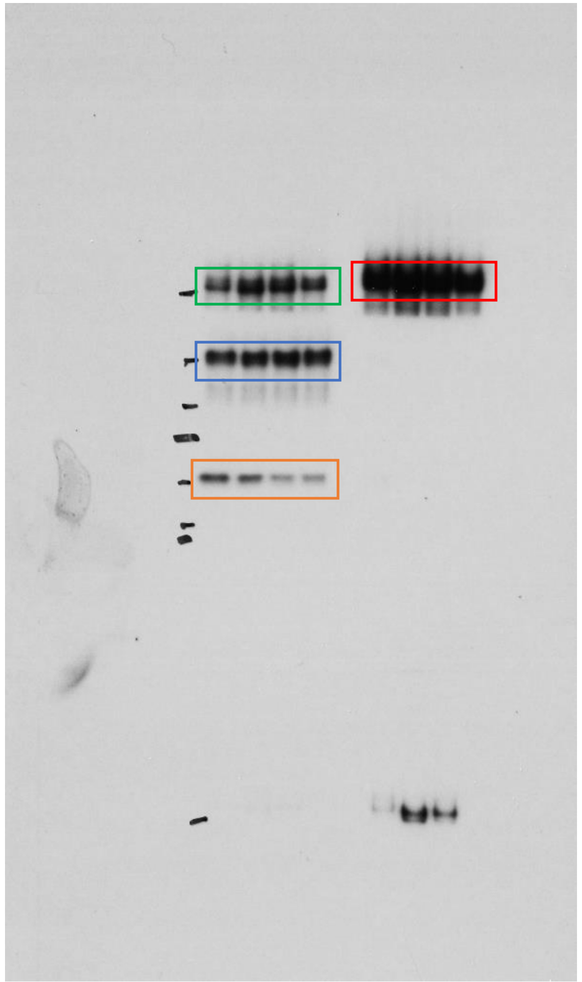

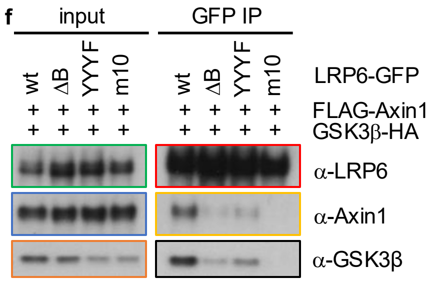

Following construct expression validation, samples were run on a single blot loaded input (left) and IP (right).

The blot was cut at 140kDa and 60kDa and probed for LRP6 (top, ~200kDa), Axin1 (middle, ~115kDa) and anti-GSK3b (bottom, 46kDa).

Gammons et al, Figure 8f

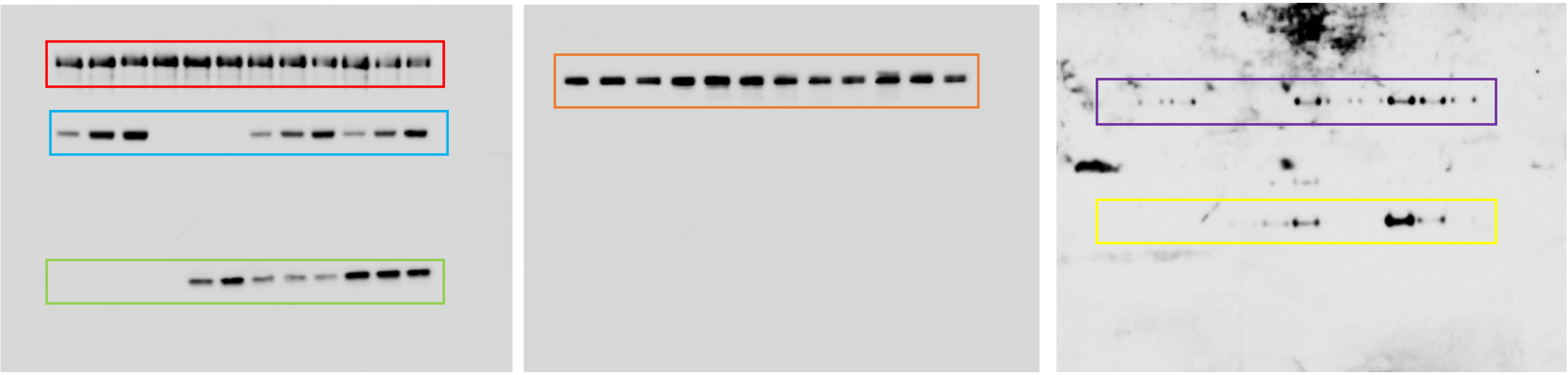

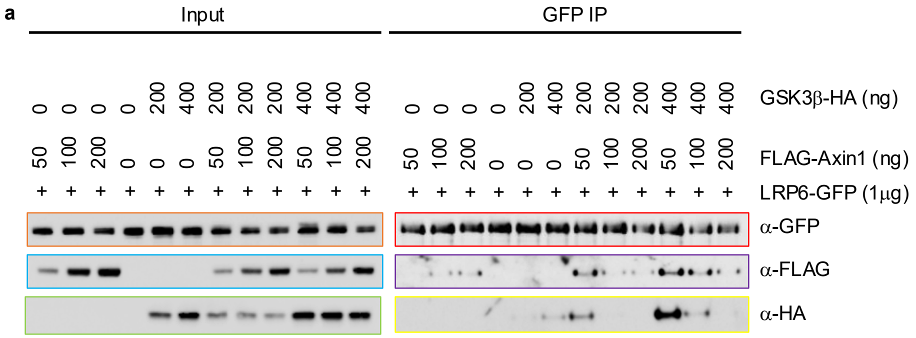

Following construct expression validation, samples were run on two blots, one loaded with input (left) and the other IP (right).

The blots were cut at 140kDa and 60kDa and probed for LRP6 (top, anti-GFP, ~200kDa), Axin1 (middle, anti-FLAG, ~115kDa) and GSK3b (bottom, anti-HA, 46kDa).

Note that the LRP6 IP (top fragment) from Blot2 was imaged with the FLAG and HA (middle and bottom fragments) of Blot1, accidently. The LRP6 input was imaged separately.

The FLAG and HA IP (blot 2) were imaged together for significantly longer that the inputs and LRP6 IP, as expected.

Gammons et al, Supplementary Figure 1a

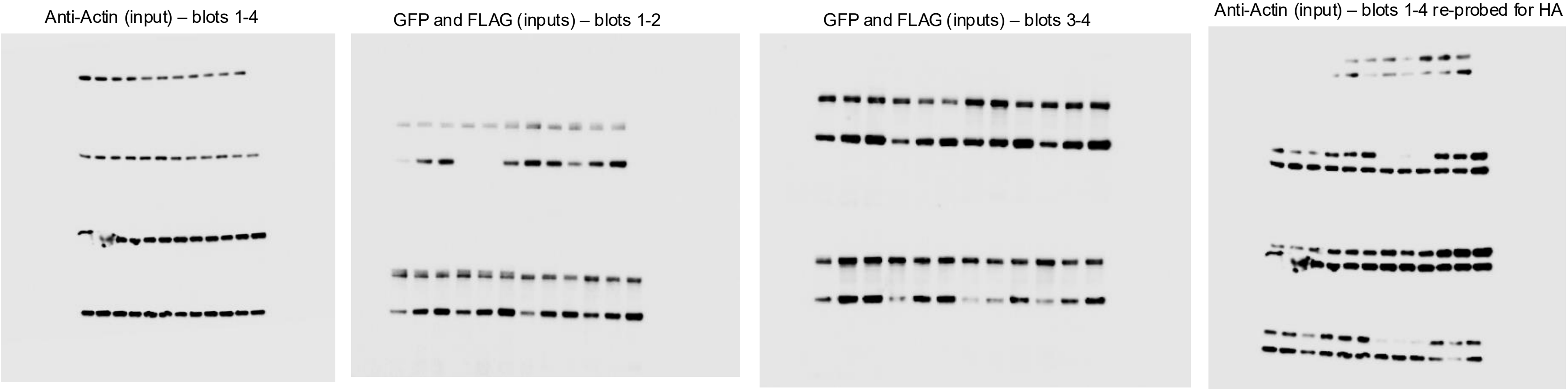

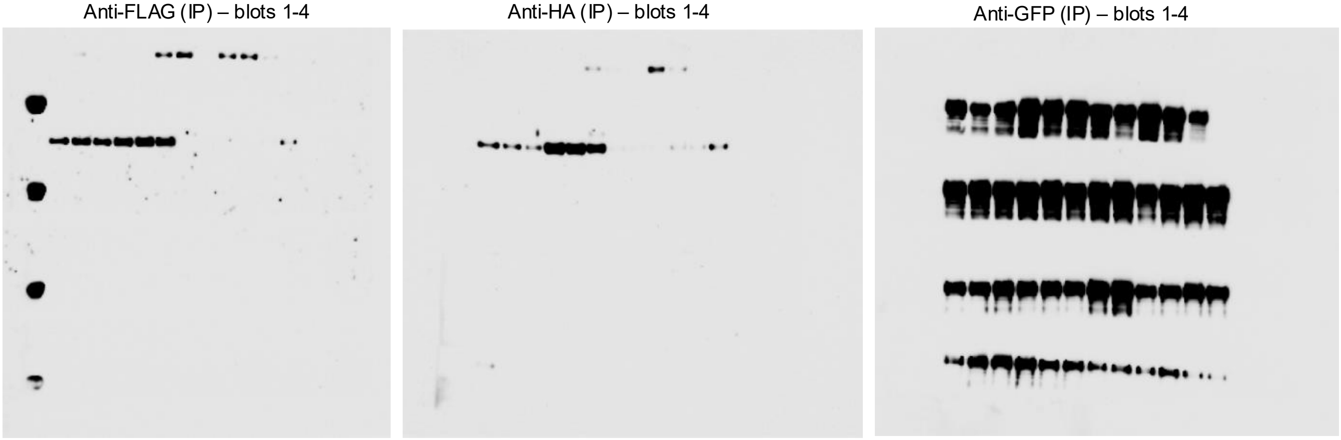

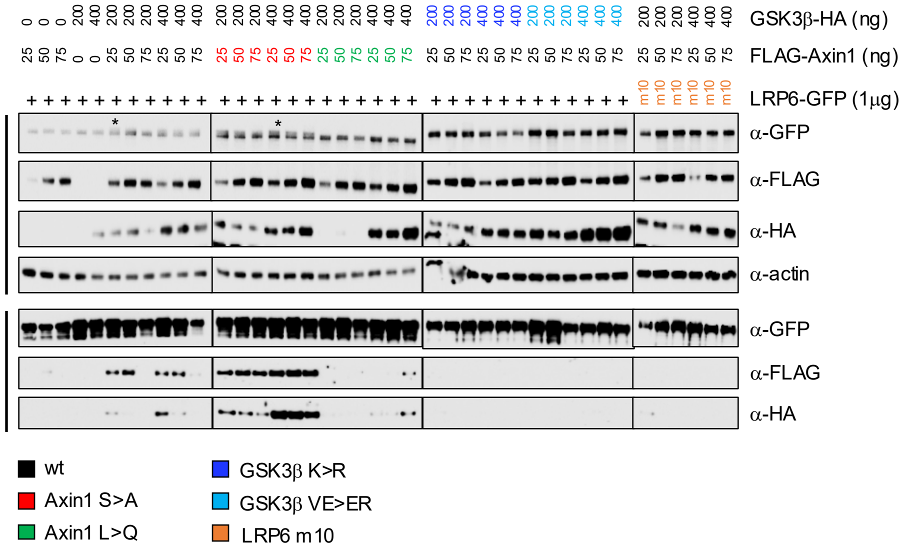

Following construct expression validation, samples were run on eight blots, four loaded with input (top) and four IP (bottom).

The blots were cut at 140kDa and 60kDa and probed for LRP6 (top, anti-GFP, ~200kDa), Axin1 (middle, anti-FLAG, ~115kDa) and GSK3b (bottom, anti-HA, 46kDa). Note inputs were first probed for actin and then reprobed for GSK3 (HA, top panel #4)

Note that rather than whole blots being imaged together the inputs for each antibody were imaged together for matched exposure times.

Gammons et al, Supplementary Figure 1b

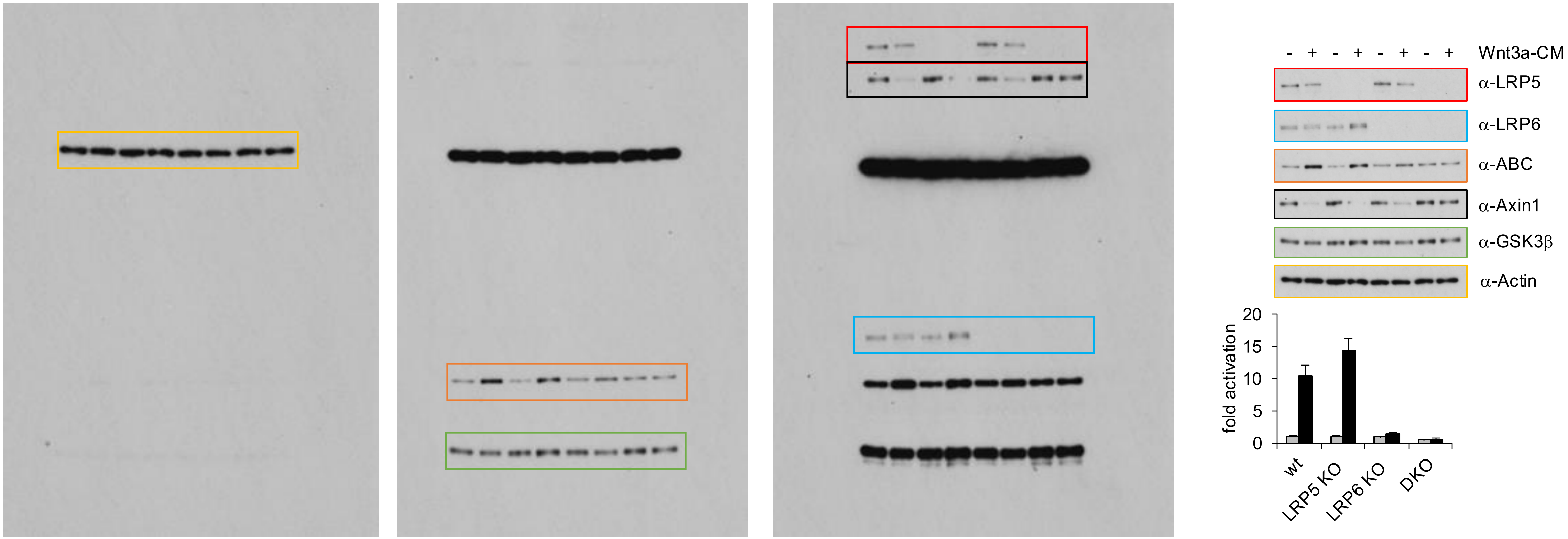

Samples were run in duplicate on two separate blots, which were cut and probed as follows:

Blot1 (top): blot was cut horizontally (at ~140kDa and ~65kDa MW) and probed for anti-LRP5 (top, ~200kDa), anti-Axin1 (middle, ~115kDa) and anti-actin (bottom, 42kDa)

Blot2 (bottom): blot was cut horizontally (at ~140kDa and ~65kDa MW) and probed for anti-LRP6 (top, ~200kDa), anti-ABC (middle, ~115kDa) and anti-GSK3b (bottom, 50kDa)

Gammons et al, Supplementary Figure 10b

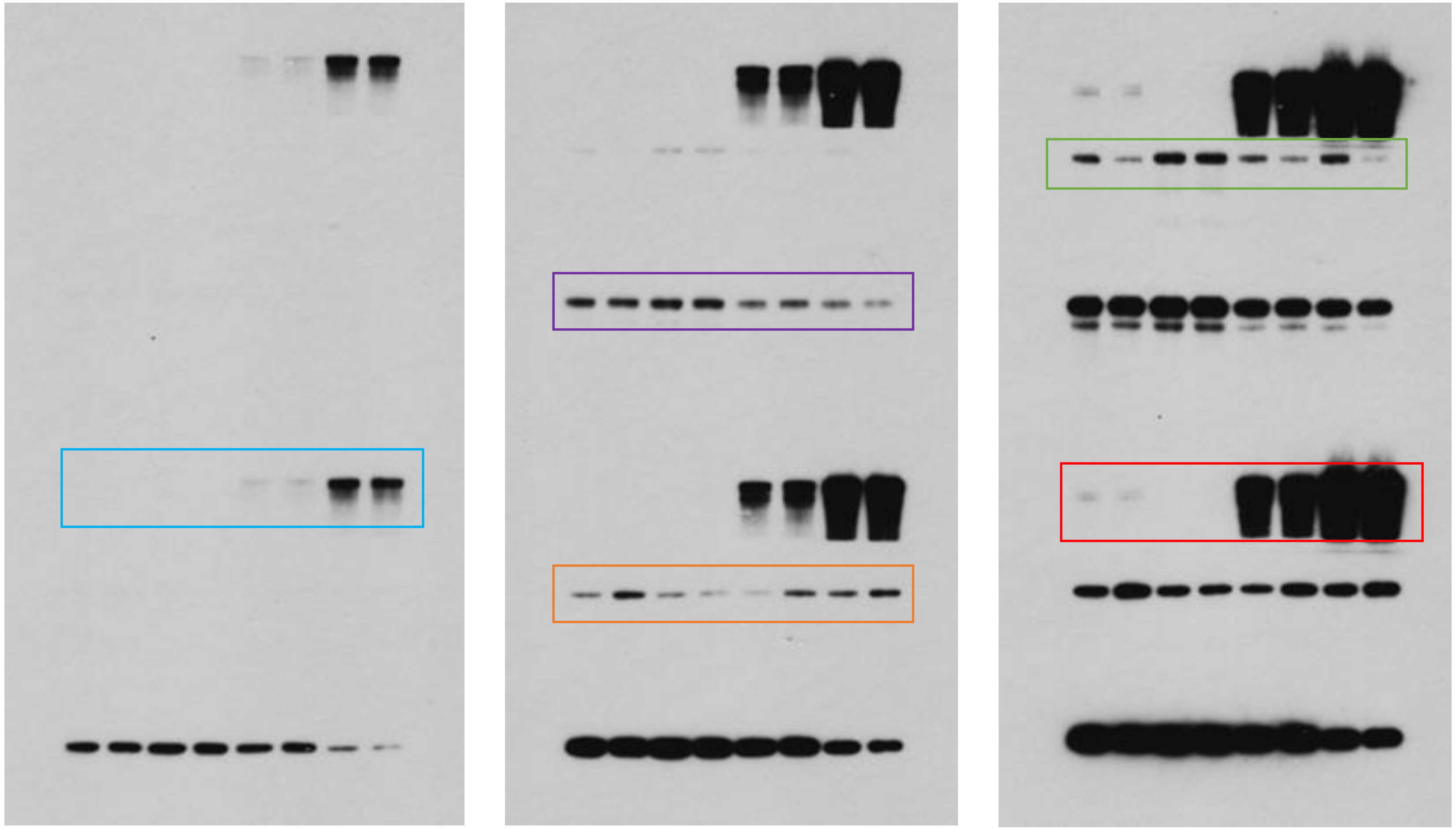

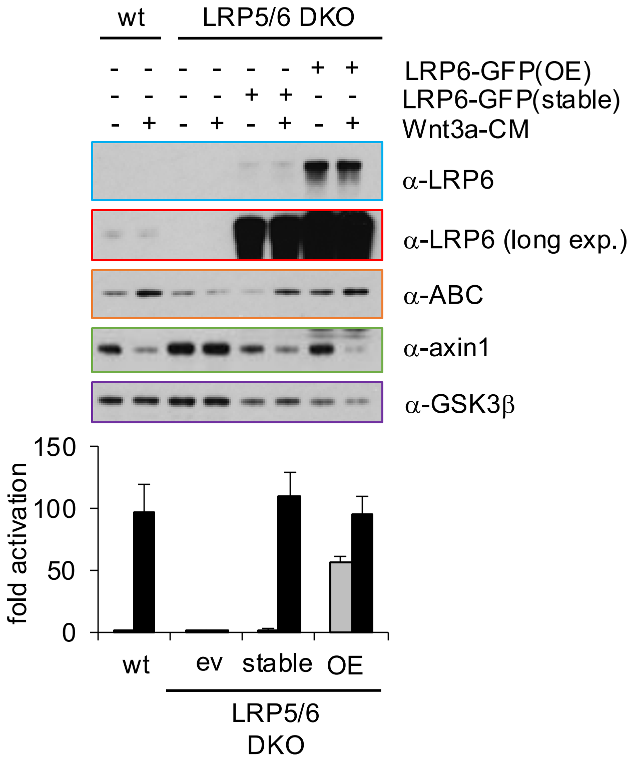

Samples were run in duplicate on two separate blots, which were cut and probed as follows:

Blot1 (top): blot was cut horizontally (at ~140kDa and ~65kDa MW) and probed for anti-LRP6 (top, ~200kDa), anti-Axin1 (middle, ~115kDa) and anti-GSK3b (bottom, 50kDa)

Blot2 (bottom): blot was cut horizontally (at ~140kDa and ~65kDa MW) and probed for anti-LRP6 (top, ~200kDa) and anti-ABC (middle, ~115kDa).

Gammons et al, Supplementary Figure 10c
